# Reduced Mechanical Tactile Stimulation Under Space Microgravity Affects Synaptic Signaling and Contributes to Neuromuscular Aging in *Caenorhabditis elegans*

**DOI:** 10.1096/fj.202600867RR

**Published:** 2026-06-16

**Authors:** Atsushi Higashitani, Je‐Hyun Moon, Jong‐In Hwang, Nahoko Higashitani, Toko Hashizume, Ahmad Aisha Abu, Kazuki Ooizumi, Ibuki Sazuka, Yoshimitsu Hashizume, Masumi Umehara, Alfredo V. Alcantara, Ban‐seok Kim, Timothy Etheridge, Nathaniel J. Szewczyk, Takaaki Abe, Jin I. Lee, Akira Higashibata

**Affiliations:** ^1^ Graduate School of Life Sciences Tohoku University Sendai Japan; ^2^ Research Center for Space Cross‐Tech Tohoku University Sendai Japan; ^3^ Division of Biological Science and Technology, College of Science and Technology Yonsei University Wonju Republic of Korea; ^4^ Human Spaceflight Technology Directorate Japan Aerospace Exploration Agency Tsukuba Japan; ^5^ Advanced Engineering Services Tsukuba Japan; ^6^ Graduate School of Engineering Kurume Institute of Technology Kurume Japan; ^7^ Ohio Musculoskeletal and Neurological Institute Heritage College of Osteopathic Medicine Athens Ohio USA; ^8^ Department of Clinical Biology and Hormonal Regulation Tohoku University Graduate School of Medicine Sendai Japan; ^9^ Department of Nephrology Tohoku University Graduate School of Medicine Sendai Japan

**Keywords:** *Caenorhabditis elegans*, mechanoreceptors, microgravity, motor neuron, muscular mitochondria, neuromuscular aging, spaceflight, synapsis, tactile stimulation

## Abstract

Although space travel is becoming more accessible, our understanding of how the space environment and microgravity (μG) affect biology, physiology, and human health remains incomplete. This study examined the effects of μG on synaptic signaling and neuromuscular aging in *Caenorhabditis elegans* . The D01 cohort, consisting of L4 larvae to young adults raised in μG, exhibited a downregulation of genes linked to synaptic signaling, dopamine response, locomotion, cuticle development, and mitochondrial metabolism. This was accompanied by altered synapse dynamics, reduced motility, and shorter body length. In μG, aged worms showed a reduction in collagen gene expression, increased abnormalities in motor neuron morphology, changes in synaptic vesicle dynamics, and a collapse of mitochondrial morphology in body wall muscles, highlighting exacerbated aging‐like phenotypes. The gentle‐touch mechanoreceptor MEC‐4 was identified as a key mediator of μG‐induced body length reduction and changes in extracellular matrix gene expression. *mec‐4* mutants did not show μG‐associated body shortening. The expression of most mechanoreceptor genes, including stretch‐activated channels *unc‐105* and *del‐1*, was downregulated under μG conditions. Notably, the expression of *tmc‐1* and *degt‐1* mechanoreceptor genes was downregulated independently of MEC‐4. Restoration of physical stimulation using culture medium with small beads in space mitigated many μG‐induced neuromuscular defects and expression alterations including those in mechanoreceptor genes. These results highlight the role of mechanical stimuli in maintaining neuromuscular integrity during spaceflight and suggest that restoring tactile input could counter health risks from reduced tactile stimulation during long‐term space missions.

## Graphical abstract

Microgravity (μG) reduces mechanical tactile stimulation in *Caenorhabditis elegans* , causing neuromuscular decline, altered gene expression, synaptic defects, and accelerated aging phenotypes. The gentle‐touch mechanoreceptor MEC‐4 mediates μG‐induced body length reduction and extracellular matrix gene downregulation. Restoring tactile input with microsphere beads in space mitigates these neuromuscular impairments and aging‐related changes, highlighting the critical role of mechanical stimuli in maintaining neuromuscular integrity during long‐term spaceflight.

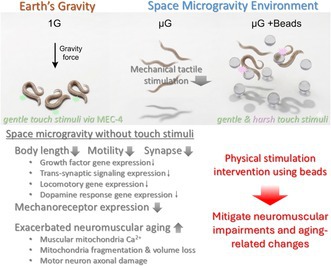

## 1 Introduction

Recent technological advancements have enabled extended habitation of humans in space for several months or more. It is well documented that microgravity (μG), which differs from Earth's gravitational conditions, induces symptoms similar to aging, such as accelerated bone loss and muscle atrophy [1, 2]. In addition to the loss of force that supports the body under μG, the redistribution of body fluids toward the head affects not only the circulatory system but also cerebral blood flow and intracranial pressure, thereby posing a recognized risk of neuroocular syndrome [3, 4, 5]. Decreases in the expression of dopamine‐related genes, such as tyrosine hydroxylase (*TH*), catechol‐O‐methyltransferase (*COMT*), monoamine oxidase A (*MaoA*), and dopamine receptor *D1R*, were observed in the brains of mice exposed to long‐term spaceflight, but these decreases were not observed in mice subjected to tail suspension, a model system of unloading in space [6, 7]. Furthermore, multi‐omics analysis of NASA's GeneLab data, which includes the biomedical profiles of 59 astronauts and transcriptional profiles of various model organisms in space, identified mitochondrial stress as a consistent central factor in spaceflight [8]. Mitochondrial and metabolic dysfunctions are also well documented to be significantly associated with neuromuscular aging [9]. Thus, understanding the mechanisms underlying spaceflight‐induced neuromuscular decline stands to not only support manned long‐term space activities but might also promote interventions that improve quality of life of elderly populations on Earth.

Microgravity in space has been shown to affect vertebrates, cultured cells, and even the invertebrate nematode *Caenorhabditis elegans* , which is about 1 mm long and contains about 1000 somatic cells but no specific gravity‐sensing cells. Exposed to μG in space, *C. elegans* exhibited decreased expression of muscle proteins, mitochondria‐related proteins, and cytoskeletal components [10]. Additionally, genes involved in cuticle development and formation were affected by both space μG and simulated μG on Earth [11]. Furthermore, a decrease in body length has been reproducibly observed in spaceflight samples, which is associated with a reduction in BMP/TGF‐β DBL‐1 signaling, a growth factor that regulates body length in *C. elegans* [10, 12, 13]. Muscle cell size and strength also decrease during spaceflight in *C. elegans* [14, 15]. These results indicate that μG exerts direct, indirect, and synergistic biological effects at various levels, including individual organisms, tissues, and cells. However, the precise mechanisms underlying these effects remain unclear.

How could the loss of gravity produce organism‐wide diverse responses in multiple tissues? Cell‐autonomous mechanisms of gravity sensing do not necessarily explain the coordinated responses to gravity observed in the tissues and organs of animals and astronauts [16]. A centrally controlled mechanism of gravity sensing, possibly through mechanosensory function, would allow coordinated changes over the lifespan of the animals. Our previous study established that in *C. elegans* , the absence of physical contact stimulation in culture under simulated μG conditions (3D clinostat) leads to a reduction in endogenous dopamine production and a decrease in the expression of *comt‐4* [17]. Notably, the intervention of physical stimulation via beads restored both gene expression and dopamine production under simulated microgravity using a 3D clinostat. Additionally, declines in *comt‐4* gene expression and endogenous dopamine levels were observed in worms cultivated in space μG [17] suggesting that diminished contact stimulation associated with the loss of gravity force of μG may affect synaptic signaling.

This study aimed to test this hypothesis through space experiments to investigate whether μG contributes to nervous system and neuromuscular aging in *C. elegans* . Additionally, we examined whether the application of physical stimuli in a μG environment could mitigate neuromuscular pathophysiologies. As part of a space experiment named “Neuronal Integrated System (NIS),” we analyzed changes in gene expression with and without intervening physical contact stimulation using wild‐type *C. elegans* and a gentle‐touch mechanoreceptor *mec‐4* deficient mutant [18, 19, 20]. Finally, we aligned gene expression changes with physiological effects of space μG with and without restoration of physical contact. It is imperative to thoroughly examine the adverse neuromuscular effects of reduced tactile stimulation associated with spaceflight, such as attenuation of neuronal transmission and exacerbated aging‐like phenotypes.

## 2 Materials and Methods

### 2.1 *C. elegans* Strains

Wild‐type N2 Bristol was used for the moving behavior analysis. Gene expression analyses used N2 and TU253 *mec‐4* (*u253*) strains, while body length measurements used N2, TU253, VC1141 *trp‐4* (*ok1605*), and CB1112 *cat‐2* (*e1112*) mutants. NM664 *jsIs37*, TG2435 *vtIs1*, CZ13799 *juIs76*, CZ333 *juIs1*, and KP1148 *nuIs25* were used to visualize neurons and synapses. ATU3301 *ccIs4251*; *aceIs1* visualizes mitochondrial morphology using mitoGFP, nuclear morphology with nucGFP and mitochondrial calcium with mito LAR‐GECO in body wall muscle cells [21, 22, 23]. ATU2301 *goeIs3*; *aceIs1* visualized cytoplasmic and mitochondrial calcium [21, 22, 23]. ATU3301, ATU2301, TG2435, and CZ13799 were crossed with *mec‐4* (*u253*) to create ATU3311 *mec‐4* (*u253*); *ccIs4251*; *aceIs1*, ATU2311 *mec‐4* (*u253*); *goeIs3*; *aceIs1*, YUW145 *mec‐4* (*u253*); *vtIs1*, and YUW146 *mec‐4* (*u253*); *juIs76*. Details about all mutant alleles and transgenes can be found in WormBase (https://www.wormbase.org/#012‐34‐5).

### 2.2 Spaceflight Experiment

**FIGURE 1:**
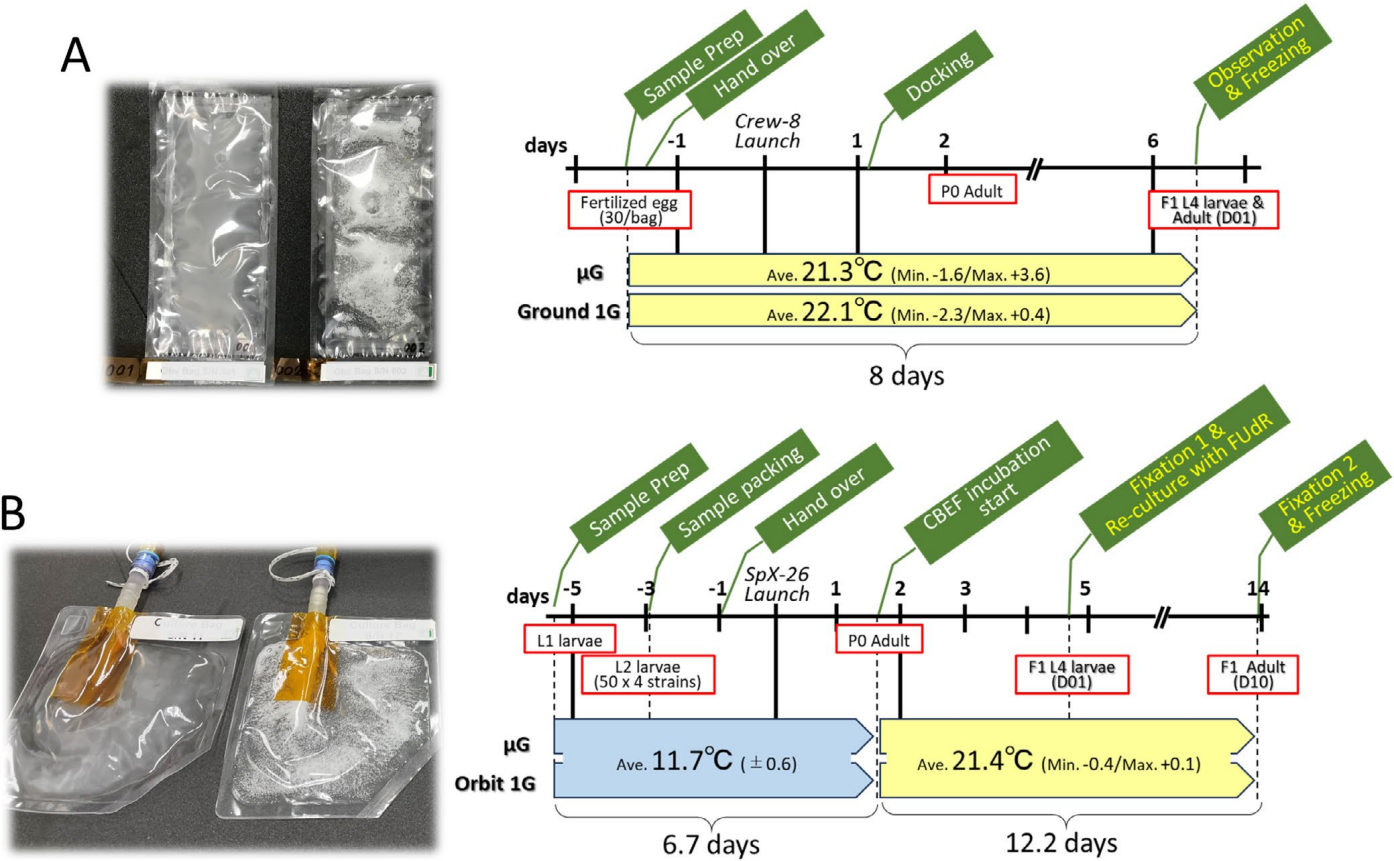
Overview of the *C. elegans* Neuronal Integrated System (NIS) experiment and cultivation status. (A) Small culture bags were used to observe F1 generation nematode motility, grown from eggs in space μG until adulthood, and to analyze gene expression after freezing. Ground 1G samples at Kennedy Space Center received identical treatment. (B) The larger bag contained 10‐day‐old adult worms grown in space with FudR added at D01 to inhibit the development of the next generation. Half of the samples were chemically fixed in orbit for histological analysis, while the other half were frozen for gene expression analysis to study the effects of μG aging. Orbit 1G samples underwent artificial 1G centrifugation on the ISS. The culture bags contained white plastic beads for contact stimulation.

The mission was conducted by the Japan Aerospace Exploration Agency (JAXA). All strains were cultured for three generations at the Kennedy Space Center laboratory (KSC; Orlando, Florida, USA) to achieve synchronized growth. Culture bags (Figure 1A) were filled with 8 mL of *E. coli* OP‐50 solution (OD_600_ = 2.) in M9 buffer with cholesterol (M9C: KH_2_PO_4_ 3 g, Na_2_HPO_4_·12H_2_O 15.1 g, NaCl 5 g, 1 mL of 1 M MgSO_4_, and 1 mL of cholesterol (5 mg/mL in ethanol) per 1 L). Static‐free polyethylene microsphere beads (WPMS‐1.00 ɸ250–300 μm, Cospheric LLC) were added at 10% [17]. Nematodes may inadvertently ingest polystyrene particles with diameters ranging from nanometers to several micrometers, resulting in their accumulation within the intestine and subsequent toxicity [24]. Consequently, in this study, we utilized materials of a size that even L1 larvae would not ingest. Thirty fertilized eggs were extracted from young adults and introduced into the solution. The bags were sealed 1.5 days before the SpaceX Crew‐8 launch and transported to ISS Kibo. After 8 days, some bags were used to observe F1 adult motility using a high‐definition camera. The remaining bags were frozen below −80°C in orbit. Space flight samples averaged 21.3°C (min −1.6°C, max +3.6°C), whereas ground control samples averaged 22.1°C (min −2.3°C, max +0.4°C). Due to the capacity limitations of the Cell Biology Experiment Facility (CBEF) [25] in the Japanese Experimental Module Kibo on the ISS, all 1G controls for the small‐type culture bags were prepared simultaneously with the samples for the on‐orbit experiments at the KSC on the ground. They were cultured in parallel under temperature conditions that closely matched the flight conditions and then frozen in a −80°C deep freezer at KSC.

Culture bags with syringe ports (Figure 1B) contained 200 L2 larvae from four GFP‐transgenic strains in 12 mL of solution. After 6.7 days of preparation and arrival at the ISS at 11.7°C ± 0.6°C, bags were incubated in CBEF under 1G centrifugation and at μG, at 21.4°C (min −0.4°C, max +0.1°C). By using this CBEF device, it is possible to accurately compare the differences between μG and arbitrary gravity created by a centrifuge in the same environment [25]. Three days later, the F1 progenies reached the L4 stage. At this point, 6 mL was transferred to a new bag containing 6 mL of M9C *E. coli* medium (OD_600_ = 4) with 5‐fluoro‐2′‐deoxyuridine (FUdR) at a concentration of 75 μM, which acts as an inhibitor of DNA synthesis to prevent the hatching of the next generation [26]. The culture was maintained for 9.5 days until reaching the D10 stage. Then, 6 mL was transferred to an empty bag and frozen below −80°C in the Minus Eighty Degrees Celsius Laboratory Freezer for the International Space Station (MELFI, JAXA) within Kibo. For the remaining 6 mL on day 4.7 (D01, L4 larvae) and day 14 (D10, aged adults), 15 mL of M9 buffer with 0.99% paraformaldehyde (PFA) was added, fixed, and stored at 4°C. The samples were returned to Tohoku University, where frozen samples were used for gene expression analysis and PFA‐fixed samples were used for GFP observation.

### 2.3 Expression Analyses

Both the space flight samples and the ground control samples from KSC were transported to our laboratory in a frozen state at the same time. Due to space constraints and handling restrictions in the space experiment, each culture condition (D01 L4 to young adult cohort) was represented by one bag (8 mL), which was cut while still frozen: approximately 2 cm (approximately 2 mL) from each end and a central section about 2 cm wide for biological triplication. These pieces were then thawed on a plastic petri dish placed on ice. Similarly, frozen samples of the D10 aged adult cohort were collected as three biological replicates from large bags. From each frozen piece, around 1000 worms (L4 to young adult cohort) were collected and washed with ice‐cold M9 buffer to remove eggs, small larvae, and debris. RNA extraction and sequencing were performed using the MGIEasy RNA Library Prep Set, MBIEasy Circularization Kit, DNBSEQ‐G400RS Sequencing Kit, and DNBSEQ‐G400 (MGI Tech).

### 2.4 Body Length Measurement of Space‐Flown Adults

Approximately 500 frozen nematodes were thawed on a plastic petri dish. Subsequently, several dozen relatively large worms, specifically adults carrying fertilized eggs, were transferred using a platinum wire onto a microscope slide under a stereomicroscope. This slide was printed with four sample wells using highly water‐repellent fluororesin‐based ink (Matsunami TF0410, Osaka, Japan) and then covered with a cover glass. The body lengths were measured utilizing a DP74 microscopic digital camera system (Olympus, Tokyo, Japan). Subsequently, the mean and standard deviation of the body lengths for the top 20 samples under each condition were calculated [12, 13].

### 2.5 Imaging and Quantitative Analysis

D01 worm movements were recorded in orbit using a high‐resolution camera, and swimming behavior frequency was calculated from videos transmitted from the JAXA Kibo ISS module. Movement was also captured using the Confocal Space Microscopy (COSMIC, JAXA), and images were downlinked. Representative images are shown in Movie S1.

Space samples were removed from plastic bags using syringes and washed with M9 buffer prior to imaging. Transgenic strains with fluorescent dopaminergic neurons, GABAergic D‐type motor neurons, mitochondria, and mitochondrial Ca^2+^ signals were observed using a scanning confocal microscope (Olympus FV10i‐ASW). The signals from mitoGFP and mito LAR‐GECO were detected using the EGFP mode (excitation at 473 nm, filter at 490–540 nm) and the Red‐Narrow mode (excitation at 559 nm, filter at 570–620 nm), respectively. All images were captured with the same laser intensity and sensitivity, and the quantification of mitochondrial Ca^2+^ levels was performed according to the method described in a previous publication [23]. The nematodes were stained with rhodamine‐conjugated phalloidin to observe the muscle fibers [21].

For CEP neurons, blebs in the dendrites were measured [22]. For D‐type motor neurons, blebs and axonal defects in the mid‐body area, including the commissural axons of VD7, DD4, VD8, and VD9, were counted (Figure S1) [27]. Blebs in four axon commissures were categorized as 1–5, 6–10, 11–15, and ≥ 16, and the proportions were plotted on a stacked graph.

Synaptic structures were examined using a Zeiss LSM 700 confocal microscope. To visualize specific presynapses of the ALM (Anterior Lateral Microtubule cells) mechanosensory neurons and the GABAergic DD/VD motor neurons, we used the transgenic strains *mec‐7p::snb‐1::GFP* and *unc‐25p::snb‐1::GFP* [28, 29]. The *mec‐7* promoter directs expression exclusively to the six mechanosensory touch neurons [30, 31], whereas the *unc‐25* promoter directs expression exclusively to GABAergic neurons including the DD/VD motor neurons [29, 32]. SNB‐1::GFP presynaptic puncta were analyzed in the dorsal nerve cord anterior to the vulva (*unc‐25p::snb‐1::GFP*) and nerve ring (NR) (*mec‐7p::snb‐1::GFP*). For ALM neurons, images were captured at a 12‐bit resolution with a maximum gray level of 4095. GLR‐1::GFP postsynaptic puncta were analyzed along the ventral nerve cord, anterior to the vulva. The number and size of fluorescent puncta were analyzed using ImageJ software.

### 2.6 Neuromuscular Aging Observation (Ground Experiment)

To inhibit egg laying during aging experiments, NGM plates with FUdR (50 μM) were covered with M9C liquid medium containing *Escherichia coli* OP‐50, and 30–50 L4 nematodes were placed on the plates. Adult worms were moved to new FUdR + M9 plates every 3 days, with dead nematodes removed daily. To observe GABAergic D‐type motor neurons, adult worms on days 1, 10, and 15 were washed with M9 buffer and observed under an Olympus BX50 microscope. Mitochondria in the body wall muscles of 3‐, 7‐, and 10‐day‐old adults were observed using scanning confocal microscopy. Samples were mounted on a 1% agarose pad and paralyzed with levamisole (motor neurons) or NaN_3_ (mitochondrial observation).

### 2.7 Imaging of Live Nematode Moving on the Ground and Simultaneous Imaging of Muscle Cytoplasmic Calcium

Using ATU2301 and ATU2311 *mec‐4* (*u253*) strains with the *goeIs3* transgene, cytoplasmic Ca^2+^ levels of adult hermaphrodites during crawling on *E. coli* OP50 plates were captured using a stereomicroscope and digital camera. Videos were converted to Royal Color and time‐lapse images using the ImageJ software.

### 2.8 Statistical Analysis

Statistical analysis was performed using an unpaired two‐tailed Student's *t*‐test or one‐way analysis of variance (ANOVA) with Tukey's post hoc analysis. Microsoft Excel, Prism software, and Chi‐square test using BellCurve were used for statistical testing. The tests are indicated in the figure legends. Statistical significance was set at *p* < 0.05. Similar letters between groups indicate no significance, whereas different letters indicate significant differences.

## 3 Results

### 3.1 Analysis of Space μG and Physical Stimuli Effects on Gene Expression in Ype‐Type *C. elegans*

**FIGURE 2:**
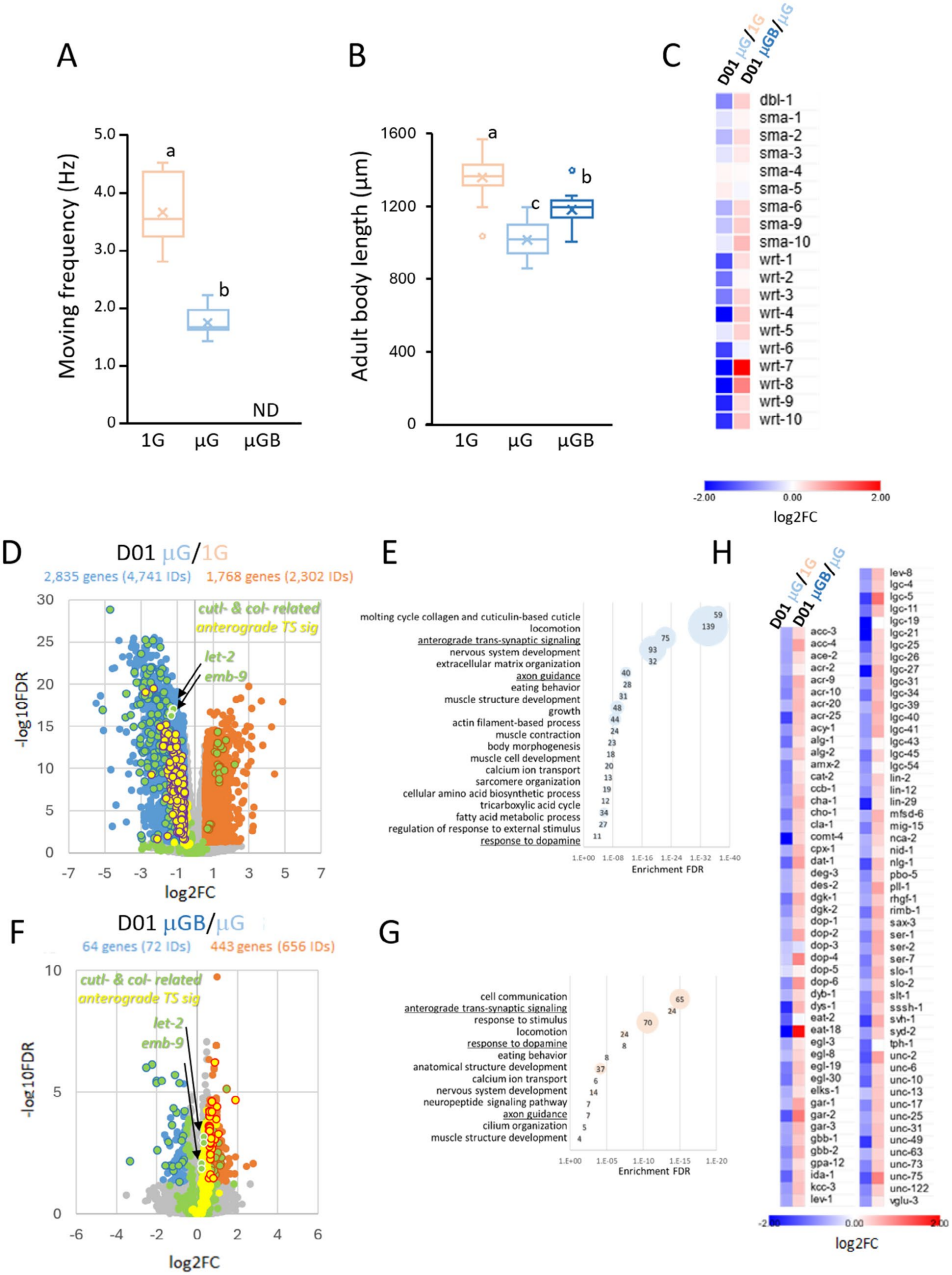
Effects of μG and bead addition under culture conditions on locomotor behavior and gene expression changes in N2 wild‐type D01. (A) D01 worms' swimming behavior frequency was calculated from video images from the JAXA Kibo module on the ISS. (B) Nematodes were cultured in bags, frozen, thawed, and the body lengths of 20 nematodes were measured. Significant differences are indicated by uppercase letters (*p* < 0.05, ANOVA with Tukey's test). (C) Heatmap analysis of log_2_(FC) values of μG/1G and μGB/μG expression of *dbl‐1*, *sma‐*, and *wrt‐* genes for body length control in *C. elegans* . (D) Volcano plot comparing gene expression by RNA sequencing of the D01 cohort at 1G and μG. Expression ratio log_2_(FC) and −log_10_FDR were plotted using three biological replicates. Blue: Decreased genes; orange: Increased genes; gray: Not significant; green: Cuticle and collagen genes; green with white outlines: *Let‐2* and *emb‐9*; yellow: Anterograde trans‐synaptic signaling and dopamine genes (purple outlines: Decreased). (E) GO enrichment analysis of 2835 genes decreased under μG/1G. (F) Volcano plot comparing gene expression in the D01 cohort under μG and μGB conditions. (G) GO enrichment analysis of 443 genes increased under μGB/μG. (H) Heat map of 101 genes with decreased μG expression related to anterograde trans‐synaptic signaling and dopamine response genes, comparing D01 μG/1G and μGB/μG expression.

During the NIS space experiment, *C. elegans* samples were launched and cultured aboard the ISS, producing an F1 generation in space μG (Figure 1A,B). Small Bag A contained L4 larvae to day‐one (D01) adults, which were frozen after the movement observation. Large Bag B was used for chemical fixation in the ISS for tissue observation. Half of the day‐10 (D10) adult worms were frozen without fixation for transcriptome analysis. To restore physical contact stimulation, we placed plastics with a specific gravity of 1.00 g/cc and a diameter of 250–300 μm into these sample bags. In this analysis of the space experiment, samples were collected from a single culture bag per condition due to sample size limitations, and the nematode population and individual animals were separated for downstream analyses. The movement frequency of worms raised in space μG was slower, and the body length became shorter than those in 1G (Figure 2A,B; Movie S1). In the bead‐added condition (μGB), worms were in constant contact with beads and therefore exhibited strong bending and extension movements; however, motility could not be quantified. However, the recovered samples showed a significant increase in body length. To investigate the molecular basis of these changes, we analyzed gene expression in the D01 cohort (L4 to young adults) grown in space μG and μGB while conducting the same analysis in a 1G environment on Earth. RNA sequencing revealed that the expression levels of certain BMP/TGF‐β DBL‐1 signaling genes, which are crucial for regulating body length, were reduced in the D01 cohort grown under μG conditions. Additionally, an improvement was observed in the μGB (Figure 2C). Similar and more pronounced expression changes occurred in the hedgehog‐like Warthog (*wrt*) gene family, which regulates molting and body morphology (Figure 2C) [33].

Volcano plot analysis showed that 4741 gene IDs (2835 genes) were significantly downregulated (FC < 2/3, FDR *p* < 0.05), while 2302 gene IDs (1768 genes) were significantly upregulated (FC > 1.5, FDR *p* < 0.05) in μG (Figure 2D: μG/1G). GO enrichment analyses revealed that the downregulated genes during μG included “molting cycle collagen and cuticle‐based cuticle (59/102),” “locomotion (139/571),” “anterograde trans‐synaptic signaling (75/262),” “nervous system development (93/427),” “extracellular matrix organization (32/58),” “axon guidance (40/142),” “tricarboxylic acid cycle (12/26),” “response to dopamine (11/35)” and others (Figure 2E, Table S1).

Upregulated genes included “positive regulation of cell cycle (18/86),” “negative regulation of cell cycle (18/99),” “negative regulation of metabolic process (68/634),” “DNA repair (37/228)” and others (Table S2). Among these GO enrichment terms, there were two opposing ones: “positive regulation of cell cycle” and “negative regulation of cell cycle.” Upon examining the genes in these GO terms, four genes, *cdk‐1*, *cye‐1*, *oma‐1*, and *oma‐2*, were present in both terms (Table S2). CDK‐1 and CYE‐1 drive cell cycle progression while also preventing incorrect transitions and maintaining checkpoints. OMA‐1/2 positively regulate meiotic cell cycle in maturing oocytes. At the oocyte‐to‐embryo transition, OMA‐1/2 repress mRNAs and degrade to end the oocyte program and enable embryonic cycles [34]. Several genes in positive regulation, including *cdc‐25.1*, *cdc‐25.3*, *gld‐3*, and *syp‐2*, are involved in meiotic entry or prophase (WormBase; https://www.wormbase.org/#012‐34‐5). Furthermore, the negative regulation genes, such as *hpr‐17*, *hus‐1*, *mus‐81*, *pch‐2*, and *wee‐1.3*, function in meiotic progression, recombination repair, and checkpoints (WormBase; https://www.wormbase.org/#012‐34‐5). Some were also enriched in the DNA repair GO term, which includes *spo‐11*. In other words, under μG conditions, the expression of genes related to somatic cells, such as those for muscle and cuticle collagen, decreased, while the expression of germline genes relatively increased. Moreover, μG may induce stress responses like the “proteasomal protein catabolic process,” which is linked to the downregulation of mitochondrial metabolic processes.

Adding beads to μG cultures (μGB) reduced altered gene expression, with 72 gene IDs (64 genes) decreased and 656 gene IDs (443 genes) increased (Figure 2F: μGB/μG). The GO analysis of upregulated genes revealed several processes that were downregulated under μG conditions, including “anterograde trans‐synaptic signaling (24/262),” “locomotion (24/571),” “response to dopamine (8/35),” “nervous system development (14/427),” and “axon guidance (7/142)” (Figure 2G, Table S3). However, the number of genes in these GO terms did not return to the levels at 1G, indicating partial recovery in the μGB.

We focused on genes (101 of 334 IDs) whose expression significantly decreased under μG related to “anterograde trans‐synaptic signaling” and “response to dopamine,” including TH/*cat‐2* for dopamine synthesis, COMT/*comt‐4* and MAO/*amx‐2* for dopamine catabolism, *dat‐1* for dopamine transport, and dopamine receptor genes *dop‐1* to *‐5* (Figure 2D,F). These included genes for serotonin and acetylcholine synthesis and receptors (*tph‐1, cha‐1, gar‐1, ‐2, ‐3, ser‐1, ‐2, and ‐7*). μGB increased the expression of most genes, as shown in the heatmap of 101 genes with Log_2_(FC) values (Figure 2H), suggesting that neuronal signaling was altered in μG and restored by contact stimulation.

### 3.2 Synaptic Vesicle Dynamics in the Nervous System in Space‐Grown *C. elegans*

**FIGURE 3:**
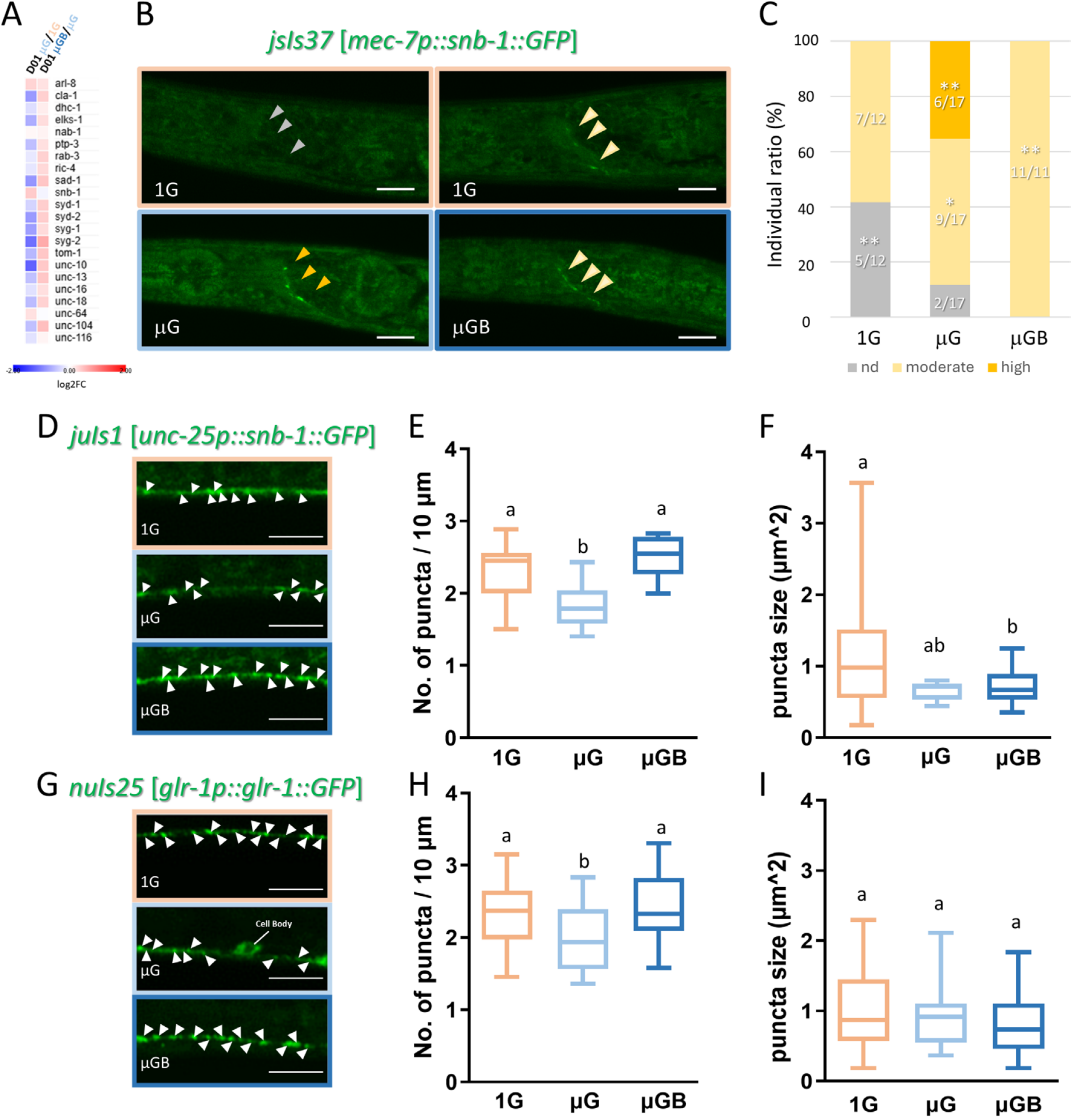
Presynaptic and postsynaptic fluorescent puncta under spaceflight μG and bead application. (A) Heatmap showing expression levels (log_2_FC) of synapse‐related genes comparing D01 μG/1G and D01 μGB/μG from RNA sequencing (L4 to young adult cohort). (B) Images of SNB‐1::GFP presynaptic puncta in ALM touch sensory neurons in the nerve ring of L4 larvae under space μG, μGB, and 1G conditions. Scale bars: 10 μm. (C) SNB‐1::GFP puncta intensity in NR synapses classified as high (< 1000), medium (300–1000), or not detected (< 300), with proportions plotted. Sample sizes: *N* = 12, 17, 11. Chi‐square test used. *p < 0.05, **p < 0.01. (D) Images of SNB‐1::GFP presynaptic puncta in GABAergic motor neurons of L4 larvae under μG, μGB, and 1G conditions. (E) SNB‐1::GFP presynaptic puncta density per 10 μm in L4 larvae (1G: *N* = 10, μG: *N* = 14, μGB: *N* = 10). (F) Synaptic puncta size (1G: *N* = 44, μG: *N* = 10, μGB: *N* = 24). (G) Images of GLR‐1::GFP postsynaptic puncta in L4 larvae under μG, μGB, and 1G conditions. (H) GLR‐1::GFP postsynaptic puncta density (1G: *N* = 25, μG: *N* = 20, μGB: *N* = 19). (I) GLR‐1::GFP puncta size (1G: *N* = 44, μG: *N* = 31, μGB: *N* = 17). Scale bars: 20 μm. One‐way ANOVA with Tukey's test; different letters indicate significant differences at *p* < 0.05.

We focused on the expression changes of 22 genes involved in neuronal vesicle trafficking, exocytosis, and synaptogenesis [35]. Heat map analysis between μG/1G and μGB/μG showed decreased expression of *liprin*/*syd‐2* and *KIF1A*/*unc‐104* (anterograde synaptic vesicle transport), *RIMS1*/*unc‐10* (synaptic membrane exocytosis), *unc‐13* (vesicle exocytosis), *cla‐1* (presynaptic active zone structure), and *syg‐1*/*syg‐2* (synaptogenesis) in the wild‐type D01 cohort in μG, and μGB restored these expressions (Figure 3A).

Next, we investigated synaptic dynamics in space‐grown L4 larvae (Large Bag B) expressing SNB‐1::GFP, a presynaptic marker that forms puncta at synapses. In worms expressing *jsIs37* [*mec‐7p::snb‐1::gfp*], synaptic puncta were visible in the NR of ALM touch neurons (Figure 3B) [36]. In space μG, 35% showed strong SNB‐1::GFP signals, 53% showed moderate signals, and 12% showed faint signals. The Space 1G environment showed moderate signals in 60% of the cases and no signal in 40% of the cases, with no strong signals. With μGB stimulation, all specimens exhibited moderate signals. Space μG appears to influence presynaptic dynamics in ALM neurons, while touch stimulation through beads counteracts these alterations.

Since locomotory behavior changed in space μG (Figure 2A), we examined whether motor neuron synapse dynamics were altered. We focused on DD/VD GABAergic motoneurons that synapse with the body wall muscles controlling movement. In worms expressing *the juIs1*[*unc25p::snb‐1::GFP*] transgene [37], synaptic puncta were visible along the GABAergic motor neurons (Figure 3D). Space μG reduced presynaptic puncta density but not size compared with the space 1G control (Figure 3D–F), indicating presynaptic changes at the neuromuscular junction. Beads μGB reversed synaptic puncta density to levels seen in 1G (Figure 3D,E).

We also investigated postsynaptic changes using GLR‐1 expression. *glr1p::glr‐1::GFP* marks postsynaptic puncta in ventral nerve cord interneurons, with changes in puncta density linked to postsynapse formation defects [38]. GLR‐1::GFP fluorescence changes when worms are isolated but is restored by mechanical stimulation [39]. Similar to SNB‐1::GFP, the density of GLR‐1::GFP puncta decreased in μG but recovered in μGB, whereas the puncta size remained unchanged (Figure 3G–I). This suggests that space μG affects synaptic gene expression and pre‐ and postsynaptic dynamics in the nervous system, with physical stimuli ameliorating these defects.

### 3.3 Gene Expression Changes in Aged *C. elegans* in Space μG (D10/D01)

**FIGURE 4:**
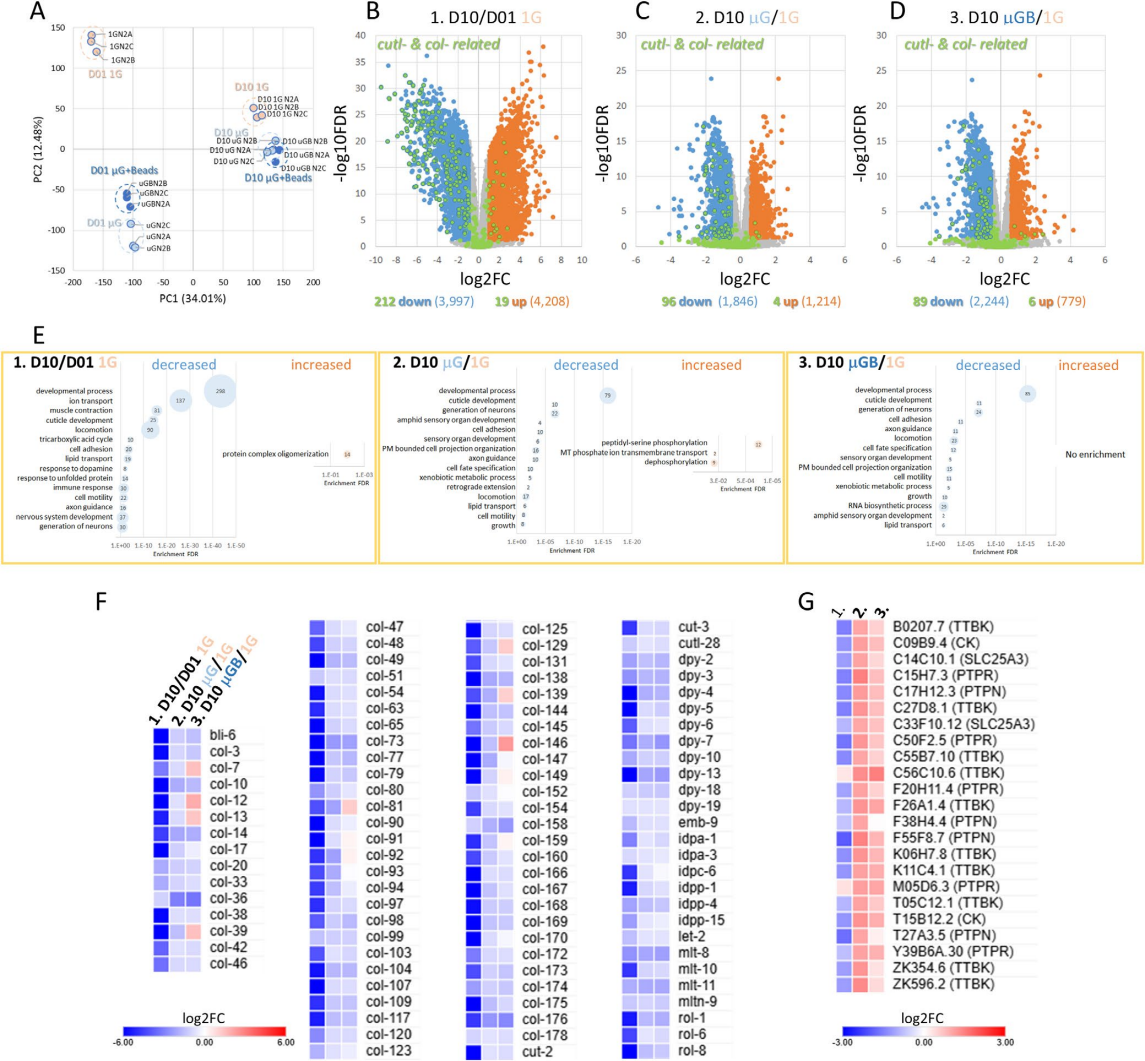
Changes in gene expression in aged worms in μG. (A) PCA analysis of gene expression with replicates in the D01 cohort (L4 to young adults) and 10‐day‐old (D10) adults under μG, μGB, and 1G conditions. (B) Volcano plot of age‐related DEGs in D10 1G/D01 1G. Blue: Decreased genes; orange: Increased genes; gray: Not significant; green: Cuticle/collagen genes. (C) Volcano plot of DEGs comparing D10 μG/D10 1G. (D) Volcano plot of DEGs comparing D10 μGB/D10 1G. (E) GO enrichment analysis of DEGs from plots (B), (C), and (D). (F) Heatmap of 96 cuticle/collagen genes decreased by μG in D10 worms, comparing D10 1G/D01 1G and D10 μGB/D10 1G. (G) Heatmap of 23 TTBK and PTPs genes enhanced by μG in D10 worms, comparing D10 1G/D01 1G and D10 μGB/D10 1G.

Previous studies on *C. elegans* spaceflight have demonstrated that μG leads to muscle atrophy and hinders neuronal debris clearance, indicating enhancement of aging‐associated features [10, 14, 40, 41]. To study the effects of space μG and aging on differentially expressed genes (DEGs) in *C. elegans* , we performed RNA‐seq analyses on D01 (a L4 to young adult cohort) and 10‐day‐old (D10) wild‐type adults treated with FUdR. D10 worms were cultured by transferring half of the F1 generation L4 larvae to fresh culture bags with FUdR (Figure 1B). PCA of gene expression, with biological triplicates under each condition, identified three clusters: the 1G group within the D01 cohort and the μG and μGB groups in D01 and D10 under all gravity conditions (Figure 4A). PC1 captured age‐related changes between D01 and D10, while PC2 represented growth environment changes at 1G and μG, with gravity change effects being most significant in the D01 cohort. Gene expression in the D01 μGB cohort shifted toward the D01 1G cohort along the PC2 axis.

To identify age‐related genes, we examined the DEGs at D01 and D10 in the 1G environment. Volcano plot analysis showed that 3997 gene IDs significantly decreased with age (less than two‐thirds, FDR *p* < 0.05), and 4208 IDs increased (more than 1.5‐fold, FDR *p* < 0.05) (Figure 4B). GO enrichment analysis revealed that genes were enriched in terms such as “cuticle development,” “cell adhesion,” “muscle contraction,” “locomotion,” “response to dopamine,” “nervous system development,” and others (Figure 4E, Table S4). These enriched GO terms aligned with those downregulated in μG/1G in D01 worms (Figure 2). Of the 348 gene IDs involved in extracellular collagen, 212 showed a significant aging‐related decrease under 1G conditions, while 19 showed an increase (Figure 4B, green dot). Collagens decrease or irregularly accumulate with age, causing various aging phenomena [42, 43]. The increased gene IDs showed enrichment only in “oligomerization of protein complexes,” with 14 gene IDs from the TNF‐alpha induced protein 1 family, containing KCTD (Figure 4G, Table S5). These mammalian orthologs are implicated in aging and neuropsychiatric disorders [44].

Comparing D10 aged worms under different gravity conditions, collagen gene cluster expression was more reduced in μG than in the artificial 1G environment of the ISS (Figure 4C; D10 μG/D10 1G). GO analysis of DEGs with decreased expression in D10 μG showed enrichment of “muscle contraction,” “locomotion,” “cuticle development” and neuron‐related terms, “response to dopamine,” “axon guidance,” “nervous system development,” “generation of neurons” (Figure 4E middle panel; Table S6). Genes highly expressed in D10 μG showed enrichment of GO terms related to “peptidyl‐serine phosphorylation” including tau‐tubulin kinase (TTBK) family, “mitochondria phosphate ion transmembrane transport” and “dephosphorylation” involved in the protein tyrosine phosphatase (PTPs) family (Figure 4C,E; Table S7). Mammalian TTBKs are implicated in neurodegenerative diseases and aging, with their overexpression inducing axonal degeneration [45].

When comparing D10 1G/D01 1G, as well as D10 μG/D10 1G, and D10 μGB/D10 1G, it was found that among the 96 gene clusters related to the cuticle and collagen, whose expression decreased with aging under 1G, expression declined further under μG, while the decrease was somewhat alleviated by μGB (Figure 4D; Table S8). In the D10 μGB group, eight collagen genes were notably upregulated (Figure 4F). Conversely, in D10 μGB, the increased expression of 23 TTBK and PTPs family genes in D10 μG was suppressed, and no significant enrichment of increased GO terms was observed (Figure 4E,G).

### 3.4 Age‐Related Changes in Neurons Under μG Conditions

**FIGURE 5:**
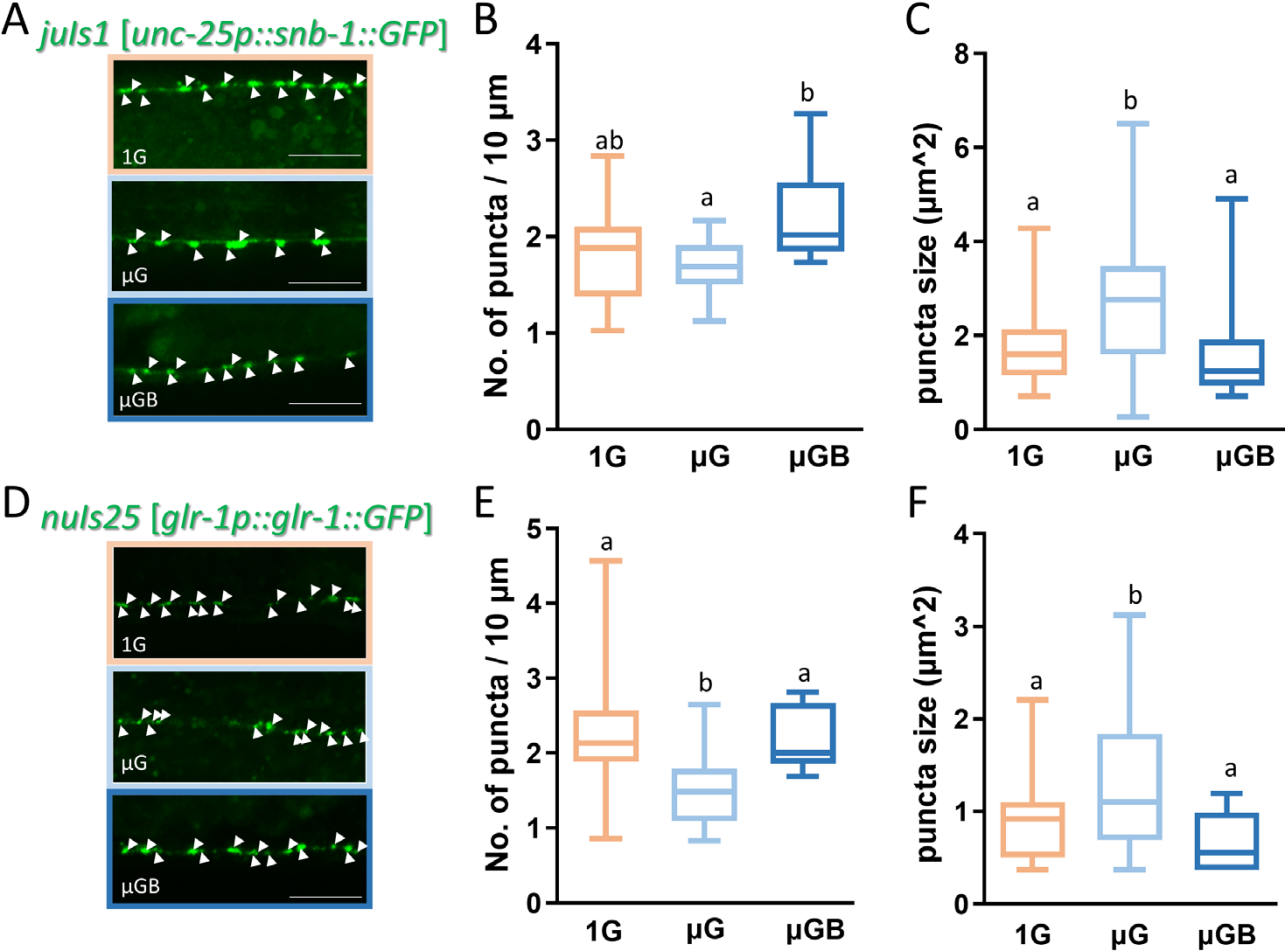
Age‐associated synaptic changes in SNB‐1::GFP and GLR‐1::GFP under space μG and μGB conditions. (A) SNB‐1::GFP presynaptic puncta images in GABAergic motor neurons of D10 aged worms under space 1G, μG, and μGB conditions. (B) SNB‐1::GFP presynaptic puncta density calculated in D10 aged worms (1G: *N* = 16, μG: *N* = 20, μGB: *N* = 14). (C) Synaptic puncta size calculated in D10 aged worms (1G: *N* = 37, μG: *N* = 37, μGB: *N* = 12). (D) GLR‐1::GFP postsynaptic puncta images in D10 aged worms under space 1G, μG, and μGB conditions. (E) GLR‐1::GFP postsynaptic puncta density calculated in D10 aged worms (1G: *N* = 42, μG: *N* = 34, μGB: *N* = 15). (F) GLR‐1::GFP postsynaptic puncta size was calculated (1G: *N* = 33, μG: *N* = 29, μGB: *N* = 12). Scale bars: 20 μm. Statistical analysis was performed using one‐way ANOVA with Tukey's test; shared letters indicate no significant difference (*p* < 0.05).

We examined the effects of μG on synapse density and size in D10 aged worms. The density of SNB‐1::GFP presynaptic puncta in DD/VD motor neurons showed no difference between D10 μG and D10 μGB when compared to D10 1G, but it was reduced in D10 μG compared to that in D10 μGB (Figure 5A,B). Conversely, the puncta size increased in the D10 μG group and was suppressed in the D10 μGB group (Figure 5C). Analysis of GLR‐1::GFP postsynaptic puncta showed decreased density in the μG group compared to the 1G controls, with beads restoring the density (Figure 5D,E). Worms aged in μG exhibited an increased puncta area, but the addition of beads restored this to 1G levels (Figure 5F). The size and intensity of these puncta correlate with vesicle accumulation and or decreased release [46, 47].

**FIGURE 6:**
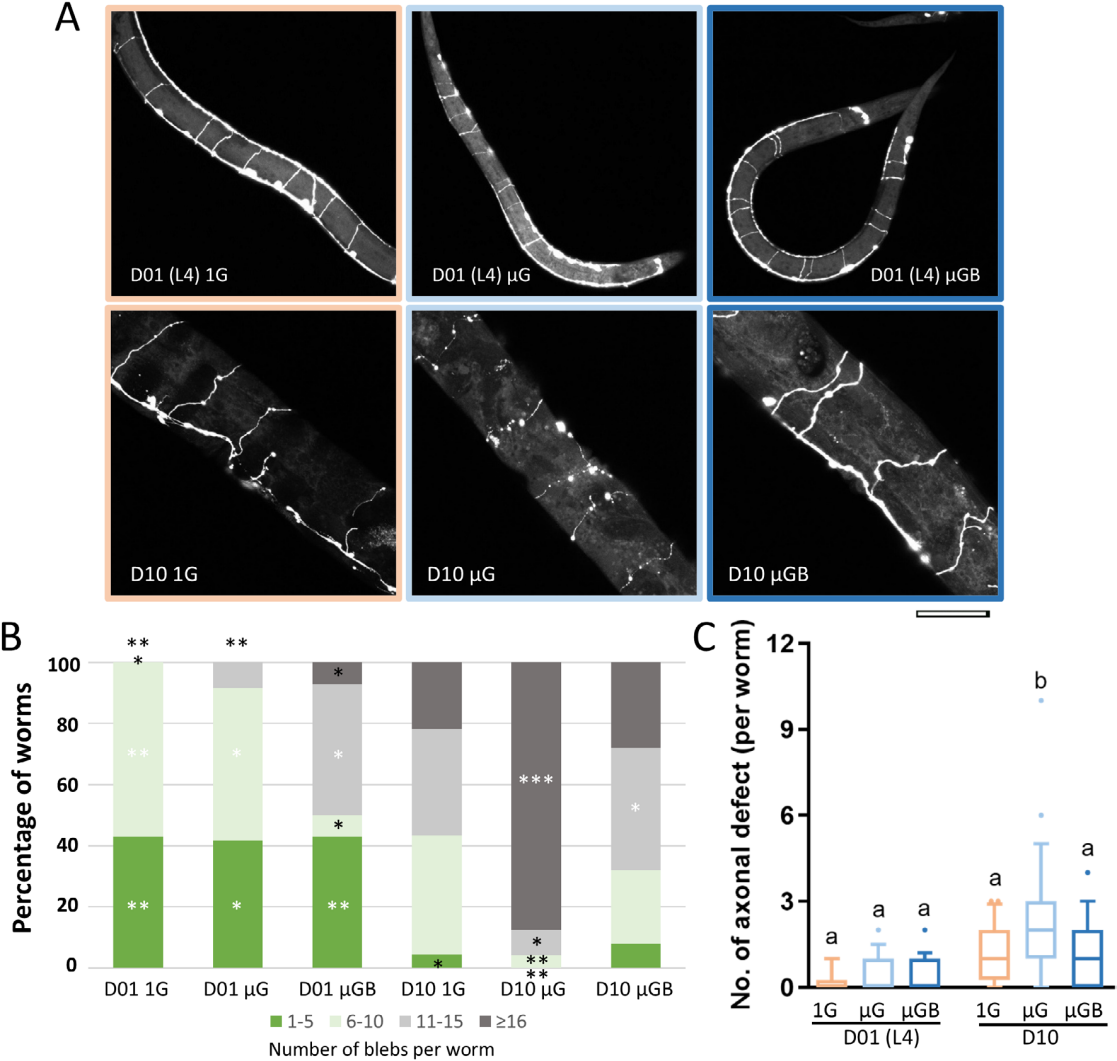
Changes in GABAergic motor neuron morphology during aging in space μG. (A) Images of SNB‐1::GFP‐labeled axon commissures of DD/VD GABAergic motor neurons (*juIs76* [*unc‐25p::Snb‐1::GFP*]) near the vulva of L4 larvae (D01) and D10 aged worms under space μG, μGB, and artificial 1G conditions. Scale bar: 50 μm. (B) Blebs in four axon commissures were categorized by damage level, and individual proportions were plotted on a stacked graph. Sample sizes from left to right: *N* = 14, 12, 14, 23, 24, and 25. Significance was tested using a 6 × 4 chi‐square test. *p < 0.05, **p < 0.01, ***p < 0.001. (C) Analysis of axonal defects, including branching, severing at the commissures, and improper rotation and fusion along the axon. The same individuals as in (B). Statistical significance was determined using one‐way ANOVA with Tukey's post hoc test. Different letters indicate significant differences at *p* < 0.05.

Given that locomotion was impaired in space and the expression of genes associated with “axon guidance” and “generation of neurons” was downregulated during aging, we monitored DD/VD motor neuron axon degeneration using *unc25p::GFP*. In the ground‐based experiment conducted at 1G, we noted axonal defects such as branching, truncation, and process merging by D10, with significant blebs forming along the axons by D15 similar to previous studies (Figure S1) [27]. In L4 larvae (D01) raised in space, the proportion of individuals with no damage in the μGB remained unchanged, while there was an increase in the proportion of individuals exhibiting a slight rise in blebs (11–15 per individual) (Figure 6). In D10 aged worms, μG increased both blebs and axonal defects compared to the 1G space control. Bead‐loaded D10 μGB restored the number of blebs and axonal defects to the levels observed in the 1G control, suggesting that physical stimulation can mitigate neuronal damage accelerated by μG aging.

Using the *dat‐1p::GFP* expression strain, we examined age‐related changes in dopaminergic sensory neuron CEPs' dendrites. In D10 aged adults, dendritic damage in CEPs was less severe than DD/VD motor neuron axonal dysfunction. The D10 μG group exhibited a slight increase in age‐related damage that was not mitigated by μGB (Figure S2).

### 3.5 Exacerbated Muscle Mitochondria Senescence in Space μG

**FIGURE 7:**
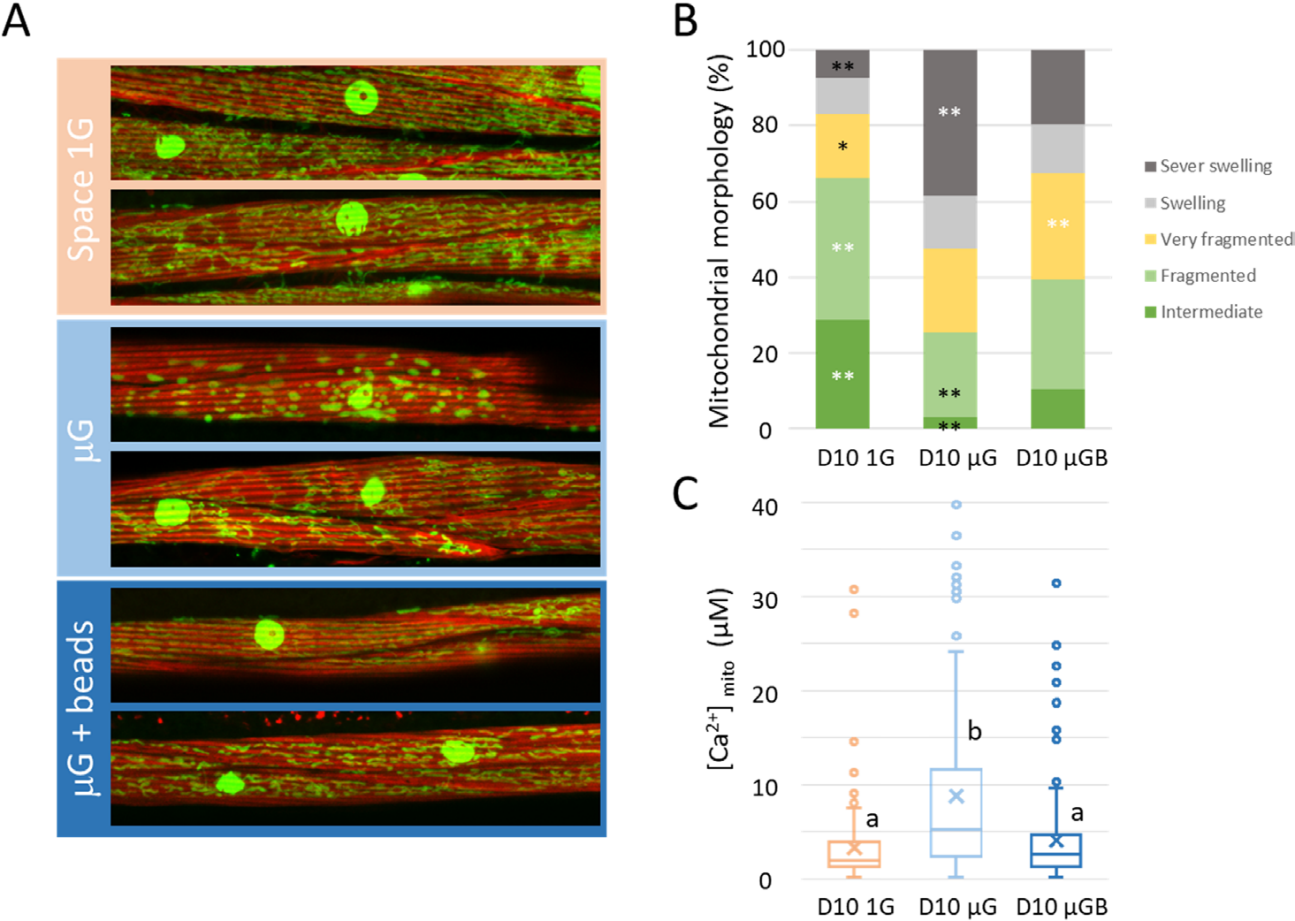
Muscle mitochondria in aged worms in space μG. (A) Images of body wall muscle cells from D10 nematode ATU3301 cultured under space μG, μGB, and artificial 1G conditions. Nuclear and mitochondrial GFP and muscle Actin filaments were visualized using rhodamine phalloidin. Scale bar: 10 μm. (B) Analysis of muscle cells with abnormal mitochondrial morphology (*n* = 250–300 cells from 40+ worms). Mitochondrial changes were classified as “intermediate,” “fragmented,” “very fragmented,” “swelling,” and “severely swollen” as shown in Figure S4. The chi‐square test was used to determine significance. *p < 0.05, **p < 0.01. (C) Analysis of mitochondrial Ca^2+^ concentrations ([Ca^2+^]_mito_) in muscle cells using mtGECO intensity. [Ca^2+^]_mito_ was calculated as described in [23]. Significance was determined using one‐way ANOVA with Tukey's test. Different letters indicate significant differences at *p* < 0.05.

*Caenorhabditis elegans* body wall muscle cells (BWMCs) contain sarcomeres and mitochondria in an observable monolayer. As aging occurs, the mitochondrial network structure fragments and its volume decreases prior to the collapse of the sarcomere [21]. Moreover, our previous studies have shown that this fragmentation and volume reduction result from the removal of sites with excessive mitochondrial Ca^2+^ accumulation, which is linked to aging or disease, through mitophagy [22, 23]. Using these transgenes *ccIs4251* (*mitoGFP*, *nucGFP*) and *aceIs1* (*mito LAR‐GECO*), we found that mitochondrial networks in L4 larvae (D01) remained unchanged in both 1G and μG environments with or without beads (Figure S3). However, D10 μG‐aged adults showed significant mitochondrial fragmentation, swelling, and volume reduction (Figure 7A,B, Figure S4). In BWMCs, 52.5% showed severely damaged mitochondria at μG, classified as Swelling and Severe Swelling, while under space 1G conditions, 16.8% of mitochondria exhibited severe damage. The addition of beads (D10 μGB) alleviated mitochondrial damage in BWMCs, decreasing the occurrence of severe damage to 32.4% (Figure 7B,C). Moreover, mitochondrial Ca^2+^ levels were significantly higher in D10 μG worms compared to D10 1G and D10 μGB (Figure 7C). In contrast, while myofibers remained nearly intact, mitochondrial aging accelerated in D10 μG (Figure 7A). This discovery indicates that the microgravity environment of space worsens dysfunction in muscle mitochondria and accelerates their removal through mitophagy, leading to signs of premature aging. As will be elaborated upon later, although the GO analysis of previous DEGs did not reveal significant enrichment, there was an observed increase in the expression of key protein genes associated with mitophagy (autophagy) in relation to both space μG and aging (see Figure 9A).

### 3.6 Effect of Mechanoreceptors on Body‐Size and Global Gene Expression Changes in Space μG

**FIGURE 8:**
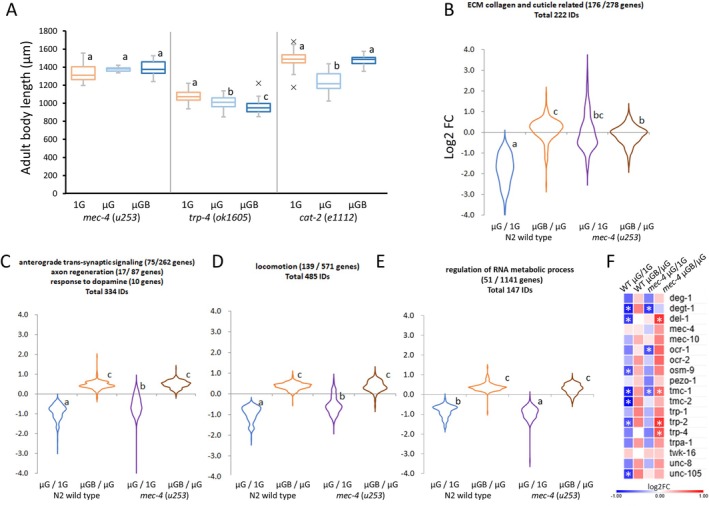
Body length and gene expression changes in mutants of mechanoreceptor genes grown under space μG, μGB, and terrestrial 1G. (A) D01 *mec‐4*, *trp‐4*, and *cat‐2* mutants were cultured, frozen, thawed, and the top 20 nematode body lengths were measured. Different uppercase letters indicate significant differences (*p* < 0.05, ANOVA with Tukey's test). (B–D) Gene expression changes of “ECM collagen and cuticle‐related” GO genes (B: 222 IDs), “anterograde trans‐synaptic signaling,” “axon regeneration,” “response to dopamine” GO genes (C: 334 IDs), “motility” GO genes (D: 485 IDs), and “RNA metabolic processes” GO genes (E: 147 IDs) were compared between wild type and *mec‐4* mutants using log_2_(FC) ratios of μG/1G and μGB/μG. (F) Heatmap analysis of the expression ratios of 18 mechanoreceptor genes between D01 wild type and *mec‐4* mutants under different gravity conditions. * indicates genes with < 0.67 or > 1.5‐fold expression at FDR *p* < 0.05.

We demonstrated that restoring physical stimulation with beads can alleviate the effects of space μG in *C. elegans* , suggesting that the lack of tactile stimulation may drive μG perception. To assess the role of touch sensation in μG signaling, we examined changes in body length in N2 wild type, *mec‐4* (*u253*), *trp‐4* (*ok1605*), and *cat‐2* (*e1112*) mutant strains. The *cat‐2* (*e1112*) mutants, which are defective in dopamine biosynthesis and are normally longer than wild type [48], showed a reduced body length under μG, similar to wild type, and the addition of beads improved their body length (Figure 8A). This suggests that dopamine depletion under μG in space [17] is not the cause of μG‐dependent body length shortening. The *trp‐4* (*ok1605*) mutant, involved in both proprioception and posterior harsh touch [49], showed reduced length under μG, with a further reduction when beads were added. However, *mec‐4* (*u253*) mutants showed no length difference between the 1G and μG environments, and bead addition had no effect (Figure 8A). These results indicate that the μG‐induced decrease in body length is mainly mediated by MEC‐4 mechanoreceptors, whereas the effect of bead addition under μG may involve TRP‐4 mechanoreceptors.

Gene expression analyses were performed on *mec‐4* (*u253*) mutants (D01 L4 to young adult cohort) raised under μG and μGB conditions and compared with those raised in a 1G environment. In the μG/1G comparison, 176 ECM collagen‐ and cuticle‐related genes that were downregulated in D01 wild type remained unchanged in *mec‐4* (*u253*) mutants, with some genes upregulated (Figure 8B, Table S9). Expression of “anterograde trans‐synaptic signaling” genes (334 IDs) and “locomotion” genes (485 IDs) in *mec‐4* (*u253*) mutants was reduced under μG, but less than in wild type (Figure 8C,D, Table S9). Expression of 51 genes involved in “regulation of RNA metabolic process” was downregulated by μG in both wild type and *mec‐4*. With beads (μGB), the expression of these three GO term genes returned to 1G levels in both strains (Figure 8C–E, Table S9), whereas collagen‐ and cuticle‐related genes showed no change in *mec‐4* (*u253*) mutants. The findings indicate that the reduction in body length under μG is primarily associated with decreased expression of ECM collagen and cuticle‐related genes due to weakened MEC‐4 mechanoreceptor tactile signaling. Additionally, there were moderate MEC‐4‐mediated alterations in synaptic signaling and locomotion genes and MEC‐4‐independent changes in RNA metabolic processes.

We wondered whether other mechanoreceptors may mediate genetic and physiological changes under μG. We evaluated the expression changes of 18 genes encoding major mechanoreceptors [50], comparing wild type and *mec‐4* (*u253*) under μG conditions, with and without beads. The expression of seven mechanoreceptor genes was significantly reduced (less than two‐thirds, FDR *p* < 0.05) under μG conditions in the D01 wild‐type cohort (Figure 8F marked with *), with most of the remaining genes showing reduced expression, except *mec‐4* and *twk‐16*. This indicates that the absence of tactile stimuli downregulates most mechanoreceptors, suggesting a downward spiral of mechanosensation. Two genes, *degt‐1* and *tmc‐1*, showed reduced expression in both wild type and *mec‐4* (*u253*) mutants in μG, indicating their independence from MEC‐4. The expression of *unc‐105* and *del‐1* was reduced only in the wild type, suggesting MEC‐4‐mediated μG‐dependent suppression. Suppression of *omc‐9*, *tmc‐2*, and *trp‐2* was marked in wild type but less in *mec‐4* (*u253*), while *ocr‐1* showed the opposite suppression. Bead‐loaded μGB restored transcriptional repression of most mechanoreceptor genes in both strains, with greater improvement in the *mec‐4* (*u253*) mutant (Figure 8F).

### 3.7 Impact of the *Mec‐4* (*u253*) Mutation on Neuromuscular Aging in a 1G Terrestrial Environment

**FIGURE 9:**
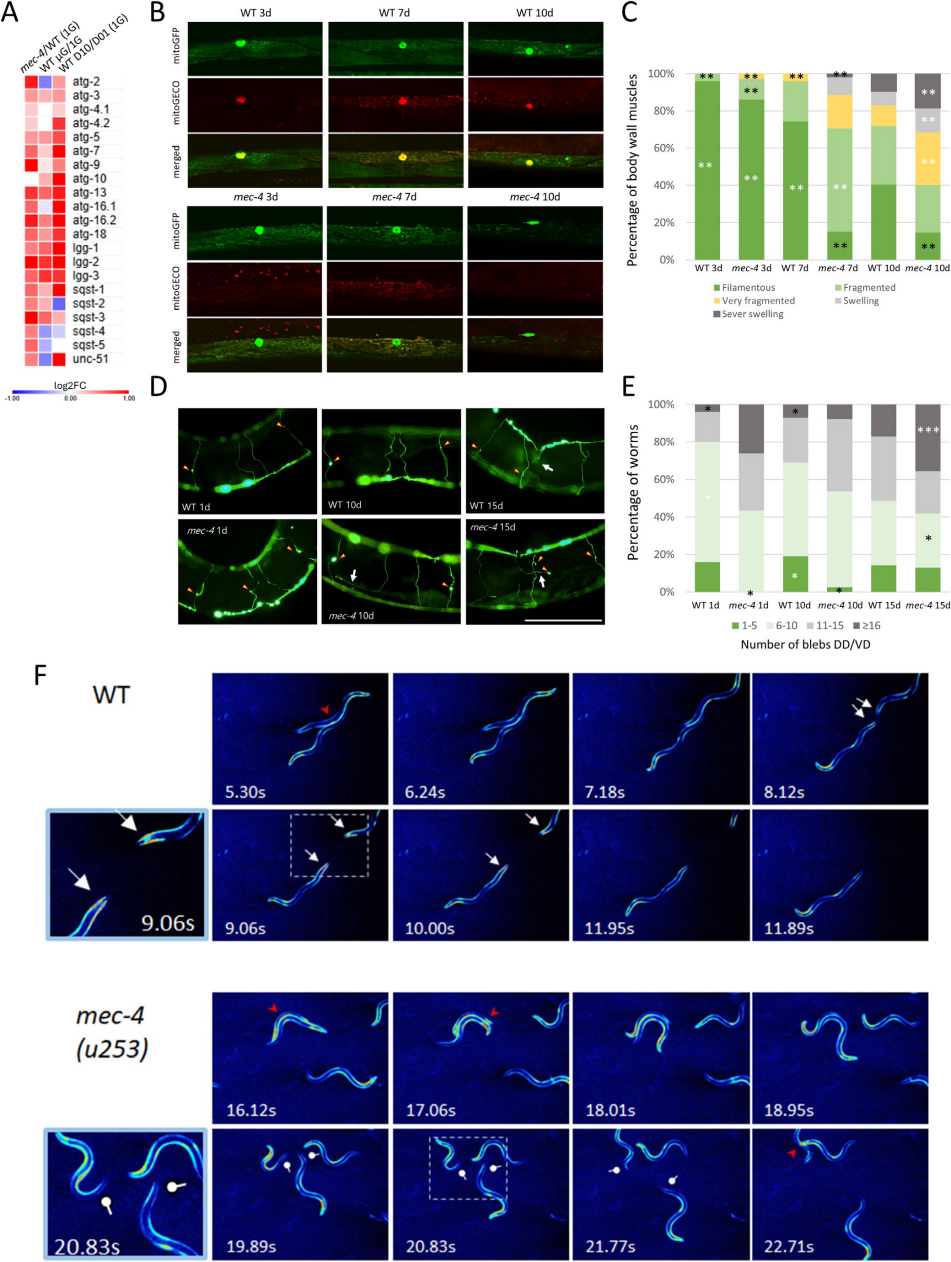
Effect of *mec‐4* mutations on neuromuscular aging and muscular calcium transients in a terrestrial 1G environment. (A) Heatmap analysis of log_2_(FC) ratio of autophagy/aggrephagy genes between D01 wild type and *mec‐4* mutants in 1G environment, plotted between N2 wild‐type D01 μG/1G and D10/D01 in 1G. (B) Images of muscle mitochondrial aging in *mec‐4* mutants vs. wild type in 1G culture. The top panels display mitoGFP and nucGFP in green, the middle panels show mito LAR‐GECO in red, and the bottom panels present the merged results. Scale bar: 10 μm. (C) Mitochondrial changes were classified as shown in Figure S4, using chi‐square test analysis (*n* = 75–250 muscle cells from 15 or more nematodes in each condition). *p < 0.05, **p < 0.01. (D) Images of age‐related changes in SNB‐1::GFP‐labeled axon commissures of GABAergic motor neurons in wild type and *mec‐4* (*u253*) mutants in 1G culture. Scale bar: 50 μm. (E) Blebs in four axon commissures were categorized according to the damage level and plotted (*n* = 25, 23, 42, 39, 35, and 31). Chi‐square test: *p < 0.05, **p < 0.01, ***p < 0.001. (F) Cytosolic calcium transients during crawling and contact stimulation in wild‐type and *mec‐4* (*u253*) adults cultured on *E. coli* OP‐50 NGM plate. Calcium concentration changes were visualized using *goeIs3* [*myo‐3p::SL1::GCamP3.35::SL2::Unc54 3′UTR* + *unc‐119*(+)] and converted using Royal Color. Sequential clips at 1‐s intervals were shown in the videos (Movie S2). The red arrowheads indicate vigorous contact events, and the white arrows/circular arrowheads indicate weak friction events when the two nematodes pass by and separate.

We studied DEGs by comparing the D01 wild‐type and *mec‐4* (*u253*) cohorts (L4 to young adult) at 1G control. GO analyses showed that downregulated genes were enriched in “molting cycle collagen and cuticulin‐based cuticle,” “ion transport,” and neuromuscular terms such as “sensory organ morphogenesis” and “neuropeptide signaling pathway” (Figure S5). These GO terms partially aligned with the genes downregulated in μG/1G in the D01 wild‐type cohort (Figure 2D, Figure S4). The upregulated genes were enriched in “reproduction,” “autophagy,” “autophagy of mitochondrion,” “aggrephagy,” “microtubule organization,” and “nervous system development” (Figure S5). Twenty‐one autophagy and aggrephagy genes were upregulated in *mec‐4* mutants compared to those in the wild type (Figure 9A), similar to the comparisons between spaceflight and terrestrial aging in wild type (Figure 9A). These results suggest that decreased touch stimuli via MEC‐4 at 1G may exacerbate aging‐like neuromuscular changes, similar to those observed under μG conditions.

We compared muscular mitochondrial collapse and DD/VD motor neuron axon degeneration between wild type and *mec‐4* (*u253*) mutants. Analysis using mitoGFP (each top panel) showed an increase in mitochondrial aging‐associated features, such as fragmentation, swelling, and volume loss, in D07 (7d) and D10 (10d) adults of *mec‐4* (*u253*) (Figure 9B,C). The red fluorescence of mito LAR‐GECO was elevated in *mec‐4* (*u253*) 7d, indicating higher Ca^2+^ accumulation. Furthermore, in *mec‐4* (*u253*) 7d, the merged green and red images revealed small punctate red dots, indicating potential mitochondrial degradation through mitophagy [23], a phenomenon observed in the wild type at 10d (Figure 9B). Similar to muscle mitochondrial impairment, axonal blebbing of DD/VD motor neurons occurred earlier in *mec‐4* (*u253*) mutants than in wild type (Figure 9D,E). However, under space μG conditions, wild‐type D10 exhibited more pronounced muscle and motor neuron aging damage than *mec‐4* (*u253*) mutants at terrestrial 1G, indicating that the effects of space μG extend beyond merely reducing MEC‐4 channel stimulation.

The *mec‐4* (*u253*) mutant lacks mechanostimuli‐activated calcium transients in ALM touch neurons during gentle touch stimuli [20]. We examined whether *mec‐4* (*u253*) affected muscle cytoplasmic calcium transients during contact stimulation compared to wild type under 1G conditions, using *goeIs3* and *aceIs1* transgenes. No differences were observed between wild type and *mec‐4* (*u253*) mutants in calcium transients within the inner contracted myocytoplasm during crawling on agar medium (Figure 9F, Movie S2). The calcium transients during vigorous contact between the nematodes were also similar (Figure 9F red arrowheads; Movie S2). However, after weak friction when worms meet and separate, calcium transients occur in the tail muscle cytoplasm of wild type but not *mec‐4* (*u253*) mutants (Figure 9F white arrows, circular heads; Movie S2). These results showed that the *mec‐4* (*u253*) mutation eliminated calcium transients in body wall muscle cells upon weak tactile stimulation and ALM touch neurons.

## 4 Discussion

This NIS space experiment was the first to uncover a new risk associated with μG: the diminished mechanical tactile stimulation due to levitation negatively impacts neuromuscular development and aging in the nematode *C. elegans* . Moreover, this risk was alleviated through physical stimulation, specifically by adding beads. Initially, we confirmed the downregulation of specific genes associated with the muscle, cytoskeleton, mitochondria, and *comt‐4*, which is responsible for the inactivation of catecholamines like dopamine, in the wild‐type *C. elegans* D01 cohort (L4 to young adults) grown under space μG conditions (Figure 2). In addition, a consistent observation was the reduced expression of TGF‐β/BMP *dbl‐1*, which positively regulates nematode body length, resulting in a shortening of body length [10, 12, 13, 14, 15, 17]. The downregulation of downstream regulators in the DBL‐1 pathway, particularly the *sma‐* and *wrt‐* genes, as well as downstream components such as *emb‐9*, *let‐2*, and *dpy‐* genes, which are ECM collagen and cuticle‐related genes, by μG also reflected the shortening of body length (Figure 2, Table S1). Interestingly, our findings also revealed that among the mechanoreceptor‐deficient mutants *mec‐4* (*u253*) and *trp‐4* (*ok1605*), cultivation in μG led to a reduction in body length for *trp‐4* (*ok1605*), similar to the wild type, whereas *mec‐4* (*u253*) did not show such a decrease (Figure 8A). In a μG environment, nematodes float freely in the liquid culture bag, unlike in 1G conditions where they sink to the bottom and receive the gentle mechanical stimulation they typically experience, which is diminished in μG. This strongly indicates that the variations in body length between 1G and μG are mainly through MEC‐4 mechanoreception.

Furthermore, under μG conditions, when beads were introduced to enhance physical contact stimulation, the nematodes exhibited pronounced bending movements to push the beads away (see Movie S1). While not completely restored, their body length showed significant recovery (Figure 2B). The improvement effects resulting from the addition of beads were consistent with experimental results obtained under simulated μG conditions on the ground using a 3D clinostat device [17]. This study also suggested that when nematodes encounter and push back against beads, it significantly induces Ca^2+^ spikes in the muscle cells, resulting in strong tactile stimulation. The subsequent question is why, under μG conditions, the addition of beads partially but significantly restored body length in the wild type, while conversely, it led to further shortening in the *trp‐4* (*ok1605*) mutants (Figure 8A). The worsening in the *trp‐4* mutants suggests that TRP‐4 is generally activated by such contact, serving not only to counteract μG‐induced shortening but also to guard against excessive stimulation from contact with beads. Notably, *C. elegans* has two parallel pathways for harsh touch mechanotransduction receptors: the TRP‐4 in PDE sensory neurons and the MEC‐10/DEGT‐1 complex in PVD and ALM sensory neurons [51, 52]. We also observed that in space μG, the expression of the *degt‐1* gene was significantly reduced in both wild type and *mec‐4* (*u253*) mutants (Figure 8F). If the expression of the *degt‐1* gene similarly decreases in *trp‐4* (*ok1605*) mutants under μG conditions, it suggests that these mutants might struggle to respond normally to harsh touch stimuli in such environments, potentially leading to impairment and further reduction in body length. Analyzing gene expressions in space μG using *trp‐4* (*ok1605*) will be necessary in the next project.

In nematodes, various mechanoreceptor molecules function across different tissues and organs. Notably, in the D01 wild type, at least seven out of the 18 mechanoreceptor genes [50], including *degt‐1* and *tmc‐1*, were significantly reduced under μG conditions (Figure 8F). In the D01 *mec‐4* mutant, while many reduction trends were similar, only these two genes showed a significant decrease. These results indicate that signaling via the MEC‐4 mechanoreceptor is reduced by μG, leading to the suppression of gene expression at other mechanoreceptors. When beads were introduced, the wild type showed a tendency toward the recovery of nearly all gene expressions, though this was not statistically significant. Conversely, in the *mec‐4* (*u253*) mutants, the restoration of expression by beads was more pronounced, with significant increases observed in four genes: *del‐1*, *tmc‐1*, *trp‐2*, and *trp‐4*. In contrast, the expression of the *mec‐4* gene itself was not significantly affected by μG or the addition of beads (Figure 8F).

As an exceptional response to the addition of beads, the *degt‐1* gene expression instead tended to decrease exclusively in the background of the *mec‐4* (*u253*) mutant. This indicates that while the μG‐induced reduction in its expression is MEC‐4 independent, the improvement observed with bead addition is dependent on MEC‐4, revealing a curious pattern of expression changes. These findings suggest that bead stimulation in μG involves not only a harsh touch response but also negative and/or complex regulations through the gentle touch MEC‐4 mechanoreceptor and other mechanoreceptors. Looking ahead, a significant advancement will be to conduct more detailed analyses of tissue‐ and organ‐specific expression at the single‐cell level using RNA sequencing methods. This approach will help explore how space flight affects various mechanoreceptor genes and their networks, including DEGT‐1 and its complexities.

The μG‐induced suppression of *unc‐105* and *del‐1* mechanoreceptor genes was reliant on MEC‐4. Both are DEG/ENaC channels, with DEL‐1 expressed in the stretch‐sensitive regions of motor neurons [53], while UNC‐105 functions as a stretch‐sensitive channel in body wall muscle cells [54]. UNC‐105 also interacts with LET‐2 type IV collagen, which forms a heterotrimer with another type IV collagen, EMB‐9, and plays a significant role in mechanotransduction and aging [42, 55]. The expression of these type IV collagen genes decreased with age and was further diminished in space μG at D01 and D10 compared to the 1G environment (Figures 2D and 4F).

Furthermore, other mechanoreceptor genes, *degt‐1* and *tmc‐1*, which were also suppressed in μG but are independent of MEC‐4. As mentioned above, in addition to its role as a harsh touch mechanoreceptor mediated by the MEC‐10/DEGT‐1 complex, recent research has revealed that DEGT‐1 also acts as a proprioceptor [56]. It is expressed in neuronal cell bodies within the pharynx (foregut, similar to the “stomach”), where it interacts with the pharyngeal basement membrane. Downregulation of *degt‐1* results in an increased pharyngeal pumping rate, potentially allowing adaptation to changes in microbial food conditions and the pharyngeal environment by reducing the shear force under μG. Similarly, in humans under μG conditions, the body cannot rely on gravity to move stomach contents into the intestine and must rely on peristalsis (rhythmic muscle contractions), making “forces from ingested material” unreliable sensory cues.

TMC‐1 is involved in alkaline pH avoidance and mechanoperception, while a recent study shows its neuroprotective role occurs through the TMC‐1–GABA–PLCβ–DAG–PKC signaling pathway [57]. Under μG conditions, the genes in this pathway—*tmc‐1*, *unc‐13*, *unc‐31*, *gbb‐1*, *gbb‐2*, *egl‐30*, *egl‐8* (*PLCβ*), and *dgk‐1*—exhibited reduced expression (Figures 2, 3 and 8). The downregulation of TMC‐1 signaling may be associated with more severe damage to DD/VD motor neurons under μG conditions than a single *mec‐4* (*u253*) mutant on the ground. These findings indicate that reduced tactile stimulation in space affects both MEC‐4 and other mechanoreceptors, such as those involved in the stretch and proprioceptive responses of the motor organs. This may potentially initiate a negative cycle in which the expression of these receptors is suppressed, leading to dysfunction in the tissues where they are expressed and accelerating senescence.

We noted not only the repression of genes related to “anterograde trans‐synaptic signaling,” “nervous system development,” “axon guidance,” and “response to dopamine,” but also changes in pre‐ and postsynaptic dynamics, along with the suppression of motor behavior in worms raised in space μG (Figures 2 and 3). For instance, space μG causes a 28% and 18% reduction in presynaptic and postsynaptic SNB‐1 puncta density, respectively, whereas beads treatment completely restores the defect (Figure 3). In aged worms, space caused an 11% and 49% reduction in SNB‐1 puncta density, respectively, and a 32.2% and 31.9% increase in presynaptic and postsynaptic puncta size, respectively (Figure 5). Treatment with beads, however, restored both puncta density and size in aged animals. The average body length of the top 20 observed in D01 wild‐type cohort decreased by 26.2% in μG compared to the 1G control but improved to a 12.6% decrease when beads were added. Contact stimuli in space (μGB) restored gene expression, synaptic dynamics, body length, and motility.

In *C. elegans* , activity‐dependent synaptic changes are mediated by transcription factors controlling synaptic genes, including *cla‐1*, *elks‐1*, *syd‐2*, and *unc‐10*, which are repressed by space μG (Figure 3A). Increased neuronal activity enhances synaptic gene expression and puncta intensity, accelerating behavioral responses [58]. One hypothesis suggests that μG and tactile stimulus loss may decrease motor neuron activity, leading to synaptic gene repression and altered synaptic dynamics. A study has shown that nematodes isolated on a standard agar medium experience reduced sensory stimulation, resulting in altered neuronal connectivity, delayed development, and decreased body length [39]. Mechanical tap stimuli during the L3 larval stage can restore both reduced responsiveness and shortened body length in adults. Furthermore, in experiments with *mec‐4* (*e1611*) mutants, which cause touch neuron degeneration and loss of touch sensitivity [18], no differences in body length were observed between isolated and grouped worms. These phenomena closely resemble the physiological changes observed in worms within μG environments.

Moreover, it was demonstrated that age‐related deterioration is exacerbated in the axons and presynaptic regions of DD/VD motor neurons, as well as in the postsynaptic regions of ventral nerve cord interneurons, when exposed to the space μG environment (Figures 6 and 7). Laranjeiro et al. reported that worms spending 5 days as adults on ISS showed hyperbranching in PVD and tactile receptor neurons, with accumulated neuronal waste products [40]. On the other hand, our NIS experiments did not reveal any morphological changes in dopaminergic CEP neurons. Neurons in *C. elegans* exhibit varying timings in aging phenotypes [27], and the mechanisms behind these differences remain unknown. Given the consistently observed pronounced functional impairments in the motor system, such as skeletal muscle atrophy, decreased muscle strength, and alterations in the activity of spinal motor neurons in both astronauts and rodents subjected to spaceflight [59, 60, 61], it is plausible that similar significant effects may have occurred in the body wall muscles and motor neurons of nematodes as well.

In the absence of any discernible physical damage, it was found that mice subjected to prolonged space flight exhibited a decline in the expression of dopamine‐related genes. In contrast, this decline was not detected in mice that were subjected to tail suspension [6, 7]. Video recordings of mice during spaceflight showed that, despite μG conditions, they maintained normal eating, sleeping, and defecation patterns [62]. However, there was decreased contact between the limbs/hairy skin and the culture box surfaces. C‐tactile fibers in the foot and hairy skin sense light touch, slow pain, and temperature and secrete oxytocin upon sensory stimulation [63]. The interaction between oxytocin and dopamine influences maternal behavior and infant brain development [64, 65]. Infant massage stimulates growth factors, oxytocin, opioids, and dopamine through C‐tactile and Aβ fibers, contributing to infant neurological development. These findings suggest that metazoan growth under μG conditions, where tactile stimulation is reduced, may significantly threaten neuromuscular development.

Space μG exacerbated the age‐related downregulation of cuticle‐ and collagen‐related genes compared to D10 in 1G (Figure 4C,F). Teuscher et al. found that cuticle collagen (col) mRNA levels decreased with age, and protein levels declined in the ECM [43]. They reported that the overexpression of *col‐10*, *‐13*, or *‐120* extended the lifespan of *C. elegans* . In addition, longevity interventions and mechanical loading increased the expression of collagen and ECM remodeling enzymes, suppressed collagen crosslinks, prevented ECM‐cell detachment, and formed a regulatory feedback loop through mechanosensitive hemidesmosomes. In our space experiments, adding beads under μG conditions suppressed space‐μG‐induced premature aging in synapses, motor neurons, and body wall muscles (Figures 5, 6, 7), and increased the expression of eight collagen genes, including *col‐13* (Figure 4F).

Beyond changes in the nervous system and the extracellular matrix, mitochondrial alterations in *C. elegans* BWMCs are associated with aging, longevity, and maximum velocity locomotion [21, 22, 66]. Our previous report identified that age‐related mitochondrial fragmentation, volume reduction, and swelling are driven by excessive mitochondrial Ca^2+^ accumulation and mitophagy [23]. In this NIS research, wild‐type D10 worms raised in space μG exhibited exacerbated aging‐like mitochondrial phenotypes, such as mitochondrial Ca^2+^ overload, swelling, and volume loss (Figure 7). The addition of beads significantly alleviated these impairments. In ground‐based experiments, the *mec‐4* (*u253*) mutants exhibited age‐related characteristics in muscle mitochondria earlier than the wild type (Figure 9B,C). Consistent with these findings, gene expression analysis revealed an increase in autophagy‐related genes from D01 to D10 (Figure 9A). These genes were also upregulated at D01 under μG conditions and in *mec‐4* (*u253*) mutants compared to the wild type at 1G. Based on these findings, it is suggested that under the μG conditions, where tactile and contraction stimuli to nerves and muscles are reduced, the mechanisms maintaining homeostasis of cytoplasmic and mitochondrial Ca^2+^ in these tissues may be compromised. In fact, in the D01 cohort, under μG conditions, genes such as Sarco‐Endoplasmic Reticulum Calcium ATPase (SARCA)/*sca‐1* and ryanodine receptor (RYR)/*unc‐68*, as well as several Na/Ca exchangers (NCX)/*ncx‐*genes, showed decreased expression, while their expression was restored (increased) by the addition of beads under μGB conditions, as observed among the “calcium ion transport” GO enrichment cluster (Figure 2E,G, Tables S1 and S3). In addition, GO enrichment analysis confirmed a decreased expression of “ion transport,” including *ncx*‐genes and *unc‐68*, in *mec‐4* (*u253*) mutants under 1G conditions on the ground (Figure S5).

On the other hand, even when beads were introduced into the μG environment during culture, most gene expressions did not revert to the levels seen in the 1G environment (Figure 2D,F). Notably, GO genes associated with mitochondrial metabolism, such as those involved in the “tricarboxylic acid cycle,” “fatty acid metabolism process,” and “amino acid biosynthesis pathway,” which were diminished in the D01 cohort raised under μG conditions, showed no significant improvement when comparing μGB/μG (Figure 2E,G). Consequently, the failure to enhance the expression of these mitochondrial metabolic genes by adding beads is believed to be the reason why the premature mitochondrial breakdown of muscle cells and axonal damage of motor neurons, which worsen in μG, cannot be fully restored to 1G levels (Figures 6B and 7B).

Multi‐omics analyses of NASA's GeneLab data have identified mitochondrial stress as a consistent central factor in spaceflight based on transcription profiles of astronauts and spaceflight mice, among others [8]. Recently, Wakasugi et al. [67] conducted a comprehensive investigation into the translational response under μG using human tissue cultures, as well as analyzing *C. elegans* spaceflight samples that we previously conducted. They discovered that mitochondrial translation decreases in μG conditions and that cell adhesion, along with downstream signaling, conveys gravitational stimuli to the translation process. A mouse hindlimb unloading study explicitly reports that markers of mitochondrial mRNA translation (*mtIF2*, *TACO1*, and *TUFM*) are significantly reduced in soleus during the course of unloading [68]. Moreover, mitochondrial calcium overload has been identified as a critical factor in unloading‐induced mitochondrial dysfunction, particularly concerning muscle atrophy and functional decline, both in rat muscle during hindlimb unloading and in human umbilical vein endothelial cells under simulated μG [69, 70]. Mair et al. [71] developed an automated heart‐on‐a‐chip platform and investigated the effects of space μG on the spaceflight‐induced contractile and mitochondrial dysfunction. Therefore, although the details of the mechanism remain unclear, it is strongly suggested that the space environment has a negative impact on mitochondria at both the cellular and individual levels due to the complex interplay of various factors.

In conclusion, the μG unloading environment in space causes rapid atrophy of bones and muscles, as observed in both spaceflight rodents and human astronauts [1, 2]. In *C. elegans* , which consists of just 1000 somatic cells and acts as an intermediate model between the individual and cellular levels in mammals, we have shown that one of the primary effects of space μG is attributed to the reduction in tactile stimulation associated with levitation, alongside mitochondrial dysfunction. This reduction affected neuromuscular development and integrity, exacerbating aging‐like phenotypes in space. However, restoring tactile input helps alleviate these pathophysiological conditions. This discovery indicates that maintaining homeostasis through tactile stimulation by gravity is crucial for ensuring human health during prolonged space missions.

## Author Contributions

A.H. designed the study and supervised the spaceflight experiment. J.I.L., T.E., N.J.S., and Ak.H. were coinvestigators. J.‐H.M., J.‐I.H., N.H., T.H., Y.H., I.S., J.I.L., and A.H. conducted various experiments. A.A.A., K.O., M.U., A.V.AJr., B.‐s.K., Ak.H. T.A. supported some experiments. A.H. and J.I.L. wrote the manuscript. All the authors approved the final manuscript.

## Funding

This work was supported by MEXT | Japan Society for the Promotion of Science (JSPS), 25H01374. Japan Agency for Medical Research and Development (AMED), AMED‐CREST (16814305), JP21zf0127001. National Research Foundation of Korea (NRF), 2021R1A2C101178312, RS‐2024‐00460066. Asian Office of Aerospace Research and Development (AOARD), FA2386‐24‐1‐4002. UKRI | Biotechnology and Biological Sciences Research Council (AFRC), BB/N015894/1, BB/P025781/1. UK Space Agency (UKSA), ST/R005737/1. HHS | NIH | National Institute of Arthritis and Musculoskeletal and Skin Diseases (NIAMS), ARO54342.

## Conflicts of Interest

The authors declare no conflicts of interest.

## Supporting information


**Table S1:** GO enrichment analysis of genes with decreased expression in the D01 wild type grown under μG conditions compared to the terrestrial 1G environment.

**Table S2:** GO enrichment analysis of genes with increased expression in the D01 wild type grown under μG conditions compared to the terrestrial 1G environment.

**Table S3:** GO enrichment analysis of genes with increased expression in the D01 wild type grown under μGB conditions compared to the μG environment.

**Table S4:** GO enrichment analysis of genes with decreased expression in the D010 wild type grown under 1G conditions compared to the D01 worms.

**Table S5:** GO enrichment analysis of genes with increased expression in the D10 wild type grown under 1G conditions compared to the D01 worms.

**Table S6:** GO enrichment analysis of genes with decreased expression in the D10 wild type grown under μG conditions compared to 1G conditions.

**Table S7:** GO enrichment analysis of genes with increased expression in the D10 wild type grown under μG conditions compared to 1G conditions.

**Table S8:** GO enrichment analysis of genes with decreased expression in the D10 wild type grown under μGB conditions compared to 1G conditions.

**Table S9:** Fold changes in gene expression of D01 wild type and *mec‐4* mutants compared between μG/1G and μGB/μG.

**Figure S1:** Age‐related axonal neurodegeneration in D‐type GABAergic neurons on the ground. (A) Diagram showing D‐type GABAergic motor neurons (green) in the *C. elegans* strain CZ13799 (*juIs76* [*unc‐25p::GFP* + *lin‐15*(+)] II) with GFP in GABAergic neurons. Dorsal side is top. The analyzed axonal commissures are indicated in red. (B) Fluorescence images showing axonal defects in GABAergic motor neuron commissures: “Join and reach” (top left), “Turn and extend” (top right), “Stop” (bottom left), and “Branched” (bottom right), marked with white arrows. Scale bar: 100 μm. (C) Analysis of axonal defects in the commissures is shown as box‐and‐whisker plots (median, interquartile range, 10–90 percentile). Different letters indicate significant differences (*p < 0.05). Sample sizes: *n* = 25, 42, and 35. (D) Images showing bleb formation in the commissural axons, marked by yellow arrowheads. Scale bar: 100 μm. (E) Damage assessment of GABAergic neurons in space‐grown *C. elegans* : blebs in four axon commissures categorized as 1–5, 6–10, 11–15, and ≥ 16, plotted as the proportion of individuals. The same individuals as in (C) were used. 3 × 4 chi‐square test for significance. *p‐value: < 0.05.

**Figure S2:** Microgravity‐induced bleb formation in dopaminergic CEP neurons of *C. elegans* . (A) Fluorescence images of transgenic *C. elegans* TG2435 (*vtIs* [*dat‐1p::GFP* + *rol‐6*(*su1006*)] V) expressing GFP in dopaminergic CEP neurons. Arrowheads show bleb formation in CEP neurons, and arrows indicate neuronal cell bodies. Scale bar: 50 μm. (B) To assess GABAergic motor neuron damage in space‐grown *C. elegans* , blebs in four axon commissures were categorized as 1–5, 6–10, 11–15, and ≥ 16, with proportions plotted on a stacked graph. Sample sizes from left to right: *n* = 19, 20, 27, 18, 17, and 25. A 6 × 4 chi‐square test was performed. *p < 0.05, **p < 0.01.

**Figure S3:** Muscle mitochondrial morphology in D01 L4 larvae grown under different gravity conditions. Representative images of body wall muscle cells from two D01 L4 larvae, ATU3301 (*ccIs4251* [*myo‐3p::GFP::LacZ::NLS* + *myo‐3p::mitochondrial GFP* + *dpy‐20* (+)] and *aceIs1* [*myo‐3p::mitochondrial LAR‐GECO* + *myo‐2p::RFP*]), cultured under space μG, μGB, and artificial 1G. The scale bar represents 10 μm.

**Figure S4:** Classification of age‐related muscle mitochondrial damage. Mitochondrial age‐related changes were categorized based on their morphology as follows: “intermediate,” characterized by a mix of an interconnected mitochondrial network and some small fragmented mitochondria; “fragmented,” predominantly featuring small fragmented mitochondria; “very fragmented,” consisting of small, round mitochondria; “swelling,” marked by sparse, small, swollen mitochondria; and “severely swollen,” identified by severely swollen mitochondria, significant loss of GFP signal, and progressive nuclear breakdown. The top panels display mitoGFP and nucGFP in green, the middle panels show mito LAR‐GECO in red, and the bottom panels present the merged results. The scale bar represents 10 μm.

**Figure S5:** Volcano plot and GO analyses of gene expression changes in *mec‐4* mutants under terrestrial 1G conditions. (A) Volcano plot analysis comparing gene expression by RNA sequencing of the DO1 cohort (L4 larvae to young adults) in wild‐type and *mec‐4* mutants at 1G. Expression ratio log_2_(FC) and −log_10_FDR were plotted using three biological replicates. Blue: decreased genes, orange: increased genes, gray: not significant, green: cuticle/collagen genes (blue/orange outlines: increased/decreased), yellow: autophagy genes (red outlines: increased). (E) GO enrichment analysis of 291 genes decreased in *mec‐4* mutants vs. wild type. (F) GO enrichment analysis of 411 genes increased in *mec‐4* mutants vs. wild type.

**Movie S1:** The movement behavior of D01 worms was recorded using confocal space microscopy (COSMIC) and a high‐resolution camera at the JAXA Kibo experimental module on the ISS, as well as using both ground‐based models. The acquired images were converted to mp4 format using Image J and MS PowerPoint software.

**Movie S2:** Imaging of live moving wild‐type and *mec‐4* (*u253*) adults and simultaneous muscle cytoplasmic calcium imaging on the ground. The acquired videos were converted to Royal Color using ImageJ and MS PowerPoint software.

## Data Availability

All RNA‐seq global gene expression data were deposited in the DDBJ BioProject (PRJDB37534). Any additional information required to reanalyze the data reported in this study is available from the lead contact upon request.
