# Supplementary figures and images for: Reduced Mechanical Tactile Stimulation Under Space Microgravity Affects Synaptic Signaling and Contributes to Neuromuscular Aging in Caenorhabditis elegans
Source: FASEB J. 2026 Jun 16;40(12):e72045. doi: 10.1096/fj.202600867RR (PMC13270498; doi:10.1096/fj.202600867RR)

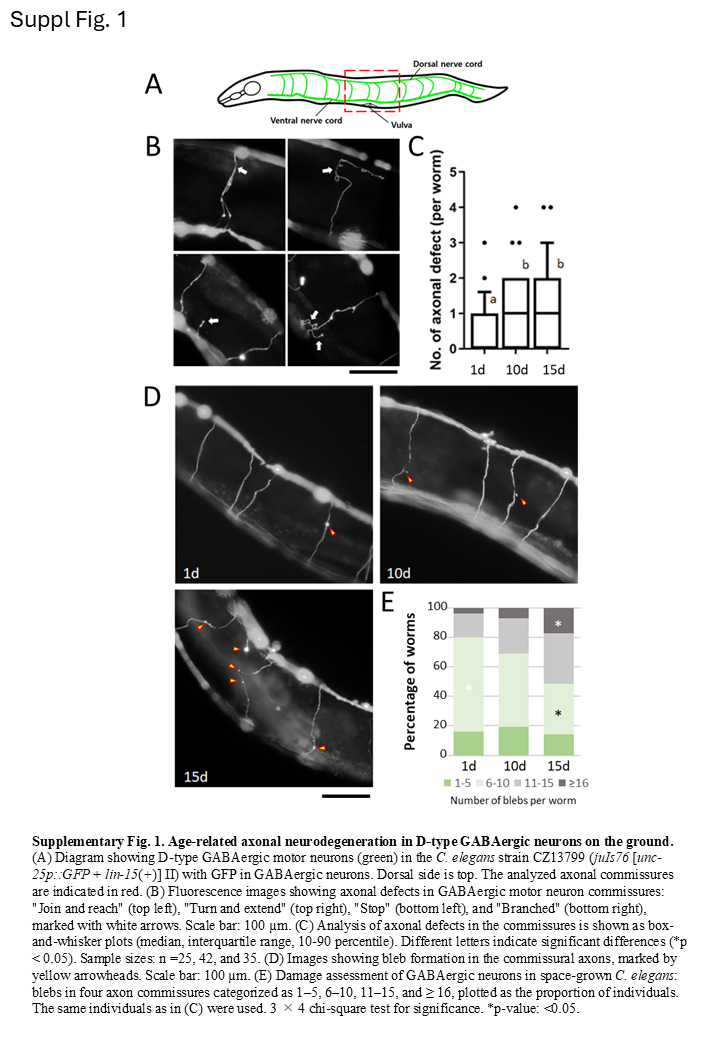

Supplement: Supplementary file 10 — Figure S1: Age‐related axonal neurodegeneration in D‐type GABAergic neurons on the ground. (A) Diagram showing D‐type GABAergic motor neurons (green) in the C. elegans strain CZ13799 (juIs76 [unc‐25p::GFP + lin‐15(+)] II) with GFP in GABAergic neurons. Dorsal side is top. The analyzed axonal commissures are indicated in red. (B) Fluorescence images showing axonal defects in GABAergic motor neuron commissures: “Join and reach” (top left), “Turn and extend” (top right), “Stop” (bottom left), and “Branched” (bottom right), marked with white arrows. Scale bar: 100 μm. (C) Analysis of axonal defects in the commissures is shown as box‐and‐whisker plots (median, interquartile range, 10–90 percentile). Different letters indicate significant differences (*p < 0.05). Sample sizes: n = 25, 42, and 35. (D) Images showing bleb formation in the commissural axons, marked by yellow arrowheads. Scale bar: 100 μm. (E) Damage assessment of GABAergic neurons in space‐grown C. elegans : blebs in four axon commissures categorized as 1–5, 6–10, 11–15, and ≥ 16, plotted as the proportion of individuals. The same individuals as in (C) were used. 3 × 4 chi‐square test for significance. *p‐value: < 0.05. [file FSB2-40-e72045-s004.tif]

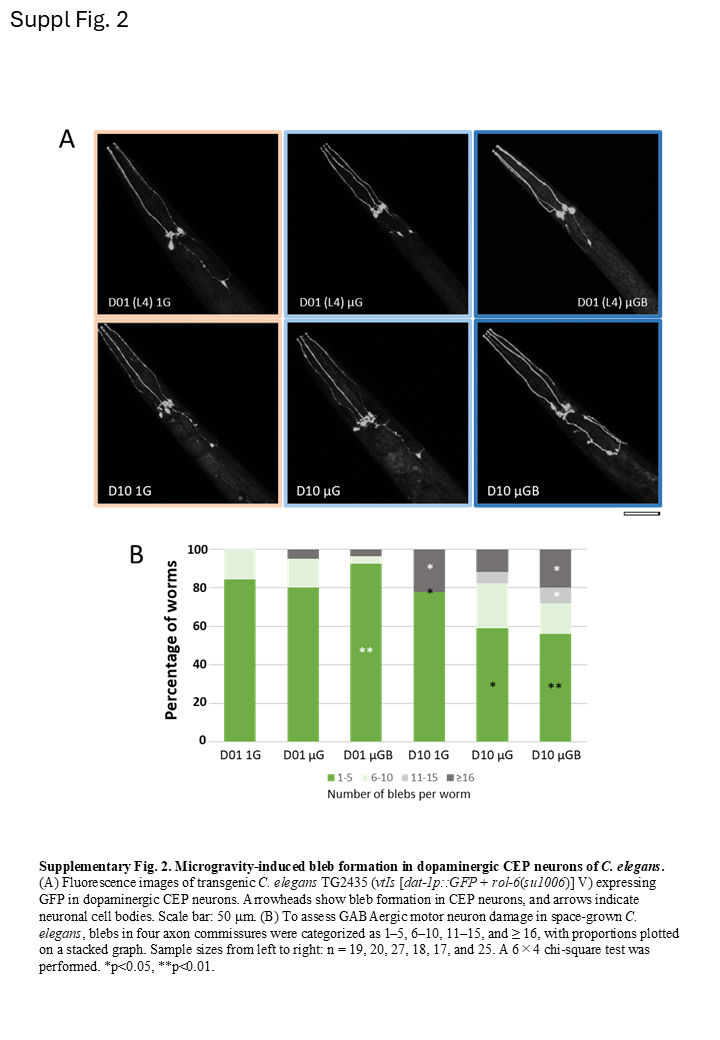

Supplement: Supplementary file 11 — Figure S2: Microgravity‐induced bleb formation in dopaminergic CEP neurons of C. elegans . (A) Fluorescence images of transgenic C. elegans TG2435 (vtIs [dat‐1p::GFP + rol‐6(su1006)] V) expressing GFP in dopaminergic CEP neurons. Arrowheads show bleb formation in CEP neurons, and arrows indicate neuronal cell bodies. Scale bar: 50 μm. (B) To assess GABAergic motor neuron damage in space‐grown C. elegans , blebs in four axon commissures were categorized as 1–5, 6–10, 11–15, and ≥ 16, with proportions plotted on a stacked graph. Sample sizes from left to right: n = 19, 20, 27, 18, 17, and 25. A 6 × 4 chi‐square test was performed. *p < 0.05, **p < 0.01. [file FSB2-40-e72045-s006.tif]

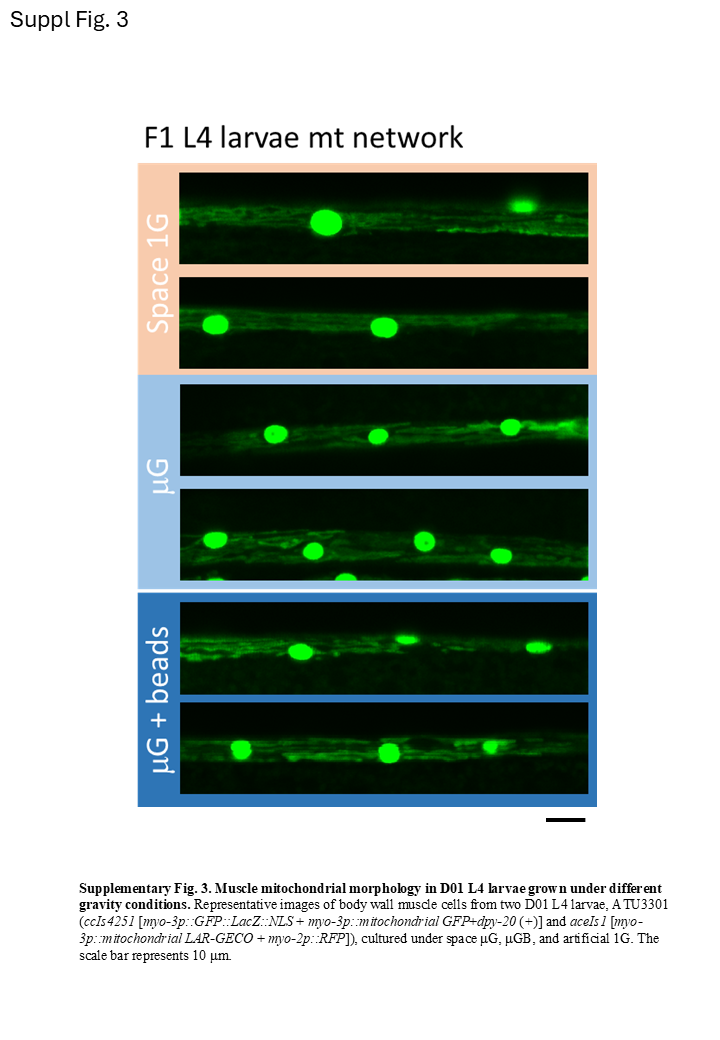

Supplement: Supplementary file 12 — Figure S3: Muscle mitochondrial morphology in D01 L4 larvae grown under different gravity conditions. Representative images of body wall muscle cells from two D01 L4 larvae, ATU3301 (ccIs4251 [myo‐3p::GFP::LacZ::NLS + myo‐3p::mitochondrial GFP + dpy‐20 (+)] and aceIs1 [myo‐3p::mitochondrial LAR‐GECO + myo‐2p::RFP]), cultured under space μG, μGB, and artificial 1G. The scale bar represents 10 μm. [file FSB2-40-e72045-s010.tif]

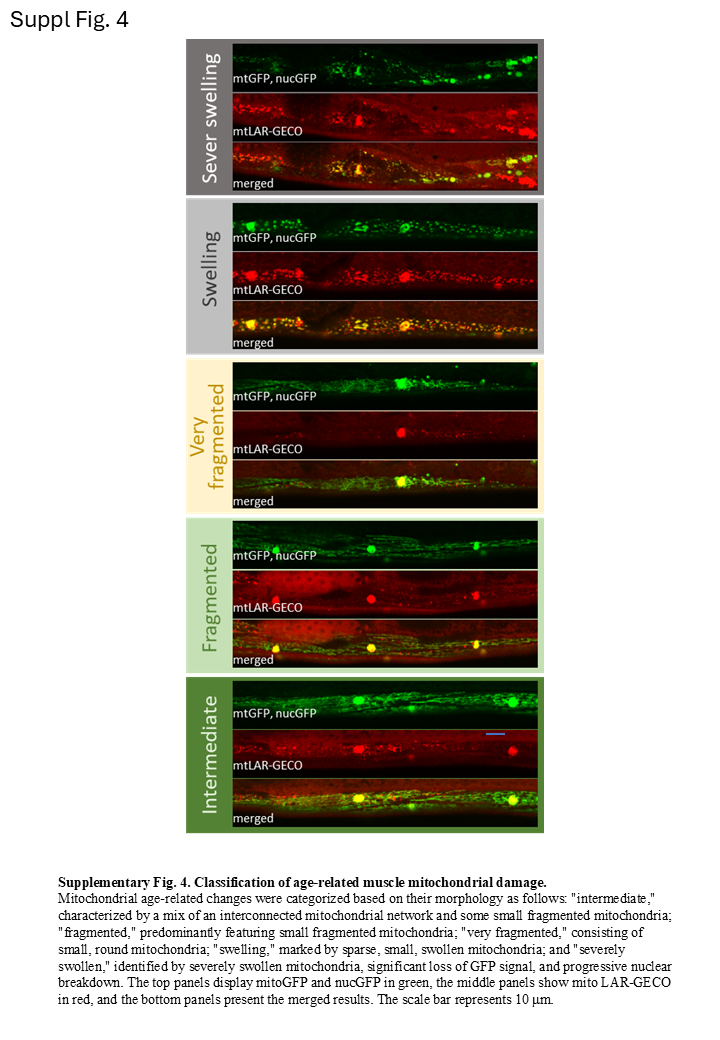

Supplement: Supplementary file 13 — Figure S4: Classification of age‐related muscle mitochondrial damage. Mitochondrial age‐related changes were categorized based on their morphology as follows: “intermediate,” characterized by a mix of an interconnected mitochondrial network and some small fragmented mitochondria; “fragmented,” predominantly featuring small fragmented mitochondria; “very fragmented,” consisting of small, round mitochondria; “swelling,” marked by sparse, small, swollen mitochondria; and “severely swollen,” identified by severely swollen mitochondria, significant loss of GFP signal, and progressive nuclear breakdown. The top panels display mitoGFP and nucGFP in green, the middle panels show mito LAR‐GECO in red, and the bottom panels present the merged results. The scale bar represents 10 μm. [file FSB2-40-e72045-s013.tif]

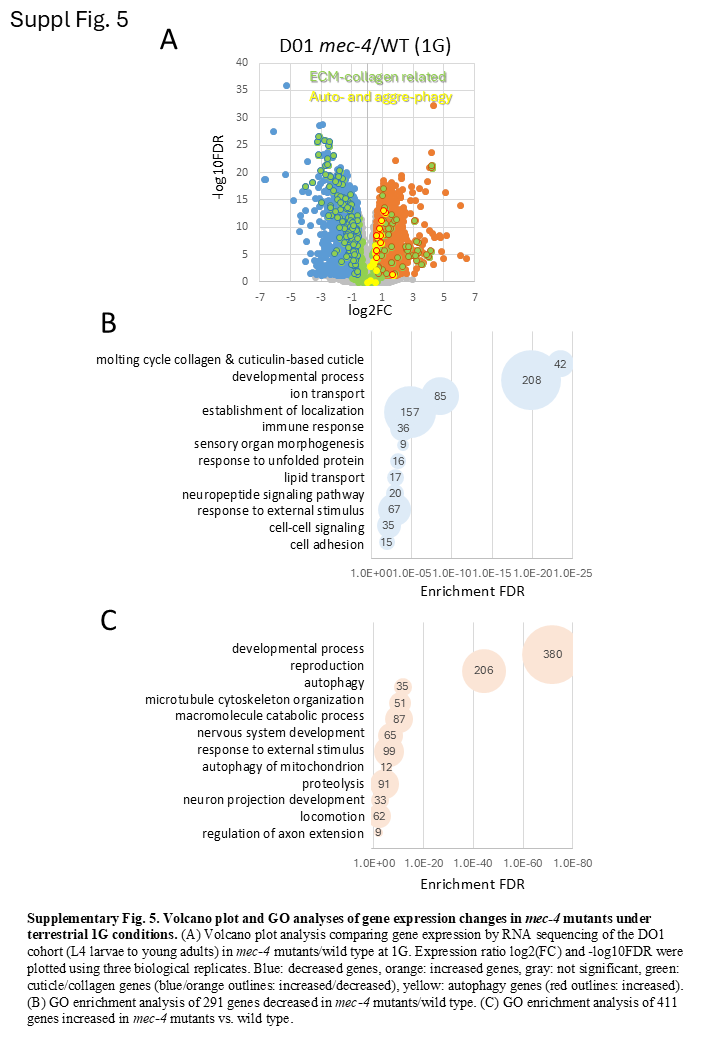

Supplement: Supplementary file 14 — Figure S5: Volcano plot and GO analyses of gene expression changes in mec‐4 mutants under terrestrial 1G conditions. (A) Volcano plot analysis comparing gene expression by RNA sequencing of the DO1 cohort (L4 larvae to young adults) in wild‐type and mec‐4 mutants at 1G. Expression ratio log2(FC) and −log10FDR were plotted using three biological replicates. Blue: decreased genes, orange: increased genes, gray: not significant, green: cuticle/collagen genes (blue/orange outlines: increased/decreased), yellow: autophagy genes (red outlines: increased). (E) GO enrichment analysis of 291 genes decreased in mec‐4 mutants vs. wild type. (F) GO enrichment analysis of 411 genes increased in mec‐4 mutants vs. wild type. [file FSB2-40-e72045-s005.tif]
